# Causal Association Between Immune Cell Traits and Risk of Multiple Malignant and Nonmalignant CNS Diseases: A Mendelian Randomization and Single‐Cell Transcriptomic Analysis

**DOI:** 10.1002/brb3.70632

**Published:** 2025-08-04

**Authors:** Shanbao Ke, Junya Yan, Baiyu Li, Xiao Feng

**Affiliations:** ^1^ Department of Oncology, Henan Provincial People's Hospital Zhengzhou University People's Hospital, Henan University People's Hospital Zhengzhou China

**Keywords:** brain disease, eQTL, immune cell trait, Mendelian randomization, scRNA‐seq

## Abstract

**Background:**

The influence of immune cell traits (ICTs) on the onset of multiple brain diseases has been previously investigated; however, it is limited by the sample size or colocalization evidence. Besides, the impact remains inconclusive.

**Methods:**

We performed a Mendelian randomization (MR) study to elucidate the causal correlation between significant ICTs and diverse brain disorders and explored the biomarkers linked to glioblastoma (GBM), a form of solid tumor, by integrating expression quantitative trait locus (eQTL) and single‐cell RNA sequencing (scRNA‐seq) analyses. The nonnegative matrix factorization (NMF) method was utilized to reclassify malignant cells into distinct cell states. Related functional analyses at the scRNA‐seq level were also performed.

**Results:**

We examined 731 ICTs across 13 brain disorders; impacts from these ICTs varied a lot across different brain diseases. Such ICTs mainly involved T/natural killer (NK) cell activation, B cell differentiation, and myeloid cell suppression or activation. Pleiotropy or heterogeneity in current results has been checked and excluded via sensitivity analyses. Specifically, colocalization analyses demonstrated protective roles of distinct ICTs in T/B/NK cell panels for amyotrophic lateral sclerosis (ALS) and GBM, while myeloid and human leukocyte antigen (HLA)‐associated traits were associated with increased risk of Alzheimer's disease (AD), and then two memory cell traits were linked to the increased risk of major depressive disease (MDD). By NMF, we identified six distinct cell states within GBM cells. Furthermore, we established an eight‐marker glioblastoma risk signature (GBRS) using scRNA‐seq and eQTL data, with higher GBRS scores observed in the NFkB cluster and EGFR cluster, indicating their highlighted aggression among malignant cells. Epigallocatechin gallate could be an effective treatment candidate targeting the EGFR cluster via markers of *SQLE* and *VCP*.

**Conclusion:**

Our findings identified causal effects of distinct ICTs on both malignant and nonmalignant brain diseases and underscored the pivotal role of neuroinflammation in their etiology. With combined evidence from eQTL and scRNA‐seq, GBM could be better characterized and managed.

## Graphical abstract

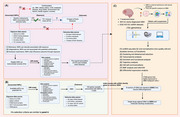

**Abbreviations**
95% CI: 95% confidence interval
AC: absolute cell counts
AC‐like: astrocyte‐like
AD: Alzheimer's disease
ALS: amyotrophic lateral sclerosis
BH: Benjamini–Hochberg
cDC: conventional dendritic cell
CNS: central nervous system
CNV: copy number variation
CSCs: cancer stem cells.
DCs: dendritic cells
DEGs: differentially expressed genes
DLB: dementia with Lewy bodies
ECM: extracellular matrix
EMT: epithelial–mesenchymal transition
eQTL: expression quantitative trait locus
FDR: false discovery rate
GBM: glioblastoma
GBRS: GBM risk signature
GBS: Guillain–Barre syndrome
GO: gene ontology
GWAS: genome‐wide association study
HVGs: highly variable genes
ICTs: immune cell traits
IDH‐wt: isocitrate dehydrogenase wild‐type
IL1β: interleukin 1β
IS: ischemic stroke
IVs: instrumental variables
IVW: inverse variance weighted
KEGG: Kyoto Encyclopedia of Genes and Genomes
logFC: log fold change
LOO: leave‐one‐out
L–R: ligand–receptor
MAPK: mitogen‐activated protein kinase
MD: molecular docking
MDD: major depressive disorder
MDSCs: myeloid‐derived suppressor cells
MES‐like: mesenchymal‐like
MFI: median fluorescence intensities
MG: myasthenia gravis
MMP: matrix metalloproteinases
MP: morphologic parameters
MR: Mendelian randomization
MR‐PRESSO: MR pleiotropy residual sum and outlier
MS: multiple sclerosis
mTOR: mammalian target of rapamycin
NK cells: natural killer cells
NMF: nonnegative matrix factorization
NOME: no measurement error
NPC‐like: neural‐progenitor‐like
OPC‐like: neural‐progenitor‐like
ORs: odds ratios
OS: overall survival
PCA: principal component analysis
PCs: principal components
PD: Parkinson's disease
PD‐L1: programmed cell death ligand 1
PI3K/AKT: phosphoinositide 3‐kinase/protein kinase B
RC: relative cell counts
RNBR: Regularized Negative Binomial Regression
scRNA‐seq: single‐cell RNA sequencing
SD: standard deviation
SNPs: single‐nucleotide polymorphisms
TAMs: tumor‐associated macrophages
TCGA‐CL: TCGA‐ classical
TCGA‐MES: TCGA‐mesenchymal
TCGA‐PN: TCGA‐proneural
TME: tumor microenvironment
TNF‐α: tumor necrosis factor‐α
UMAP: uniform manifold approximation and projection
UMI: unique molecular identifier
VEGF: vascular endothelial growth factor

## 1 Introduction

Neurological and psychiatric disorders represent a heterogeneous group of chronic conditions characterized by cognitive impairment, motor dysfunction, emotional dysregulation, and sensory disturbances (Alchalabi and Prather 2021; Lima Giacobbo et al. 2019). These disorders include neurological conditions such as Alzheimer's disease (AD), Parkinson's disease (PD), dementia, multiple sclerosis (MS), stroke, and epilepsy, as well as psychiatric conditions like schizophrenia and major depressive disorder (MDD), which collectively contribute significantly to global morbidity and mortality (Feigin et al. 2019; COVID‐19 Mental Disorders Collaborators 2021; GBD 2019 Mental Disorders Collaborators 2022). The etiology of these disorders arises from a complex interplay of intrinsic genetic predispositions and external environmental stressors. Numerous modifiable risk factors have been identified as critical for reducing the incidence and progression of brain disorders. These include the adoption of healthy lifestyle practices—such as regular physical activity, robust social engagement, balanced nutrition, and optimal sleep hygiene—as well as the avoidance of deleterious substances (e.g., alcohol and tobacco) and effective management of medical comorbidities, including hypertension, hyperlipidemia, and diabetes (Alchalabi and Prather 2021; COVID‐19 Mental Disorders Collaborators 2021; GBD 2019 Mental Disorders Collaborators 2022; Logan and McClung 2019).

The immune system is pivotal in maintaining brain homeostasis, enhancing resilience, and preserving brain reserve. Growing interest in neuroimmune interactions underscores their relevance across a broad spectrum of neurological and psychiatric disorders (Lima Giacobbo et al. 2019; Furman et al. 2017; Pape et al. 2019). The components of the innate immune response mainly include monocytes, macrophages, neutrophils, dendritic cells (DCs), mast cells, and natural killer (NK) cells (Heneka et al. 2014; Hemmer et al. 2015). On the other hand, the adaptive immune response involves B cells and T cells (Hemmer et al. 2015). While previous investigations have delineated associations between various immune cell traits (ICTs) and the risk of dementia (Y.‐R. Zhang et al. 2022; Lewis and Knight 2021; Miwa et al. 2016), PD (Jensen et al. 2021; Bottigliengo et al. 2022; Phongpreecha et al. 2020), AD (Singh 2022; Wendeln et al. 2018), MS (Thompson et al. 2018; Hemmer et al. 2015), stroke (Karim et al. 2020), schizophrenia (Campeau et al. 2022; Michel et al. 2012), and MDD (Colasanto et al. 2020; Wainberg et al. 2021), the majority of these studies have tended to focus on individual neurological or psychiatric disorders. These studies are often limited by small sample sizes and insufficient statistical power, which hinder the precise quantification of associations and restrict their clinical applicability. Furthermore, inconsistent findings complicate robust interpretation, impeding the derivation of definitive conclusions. Beyond neuroimmune interactions in neurological and psychiatric conditions, the central nervous system (CNS) plays a dual role in orchestrating ontogenesis and oncogenesis, significantly influencing cancer progression—a relatively underexplored domain in neuroscience (Winkler et al. 2023). Specifically, CNS–cancer interactions modulate oncogenesis, proliferation, invasion, metastasis, treatment response, resistance, tumor‐associated inflammation, and suppression of antitumor immune responses (Winkler et al. 2023; Monje et al. 2020; Venkataramani et al. 2019). A deeper understanding of cancer neuroscience could unlock novel therapeutic avenues. Glioblastoma (GBM), the most common primary brain tumor, is characterized by marked intratumoral heterogeneity, a poor prognosis, and limited therapeutic options beyond temozolomide (Stupp et al. 2005). Indirect neural influences on the immune system and stromal cells within the tumor microenvironment (TME) are prevalent across malignancies. Consequently, identifying ICTs that promote or inhibit GBM progression could provide critical insights into its underlying biological mechanisms.

In this study, we employed Mendelian randomization (MR) to elucidate the causal relationships between ICTs and a spectrum of brain disorders. Addressing a frequently overlooked aspect of CNS research, we integrated single‐cell RNA sequencing (scRNA‐seq) and expression quantitative trait locus (eQTL) data to develop a molecular signature strongly associated with GBM progression. Our findings provided robust evidence of a causal link between ICTs and brain pathologies, offering novel insights into disease mechanisms and potential therapeutic strategies.

## 2 Materials and Methods

### 2.1 MR Study Design

**FIGURE 1:**
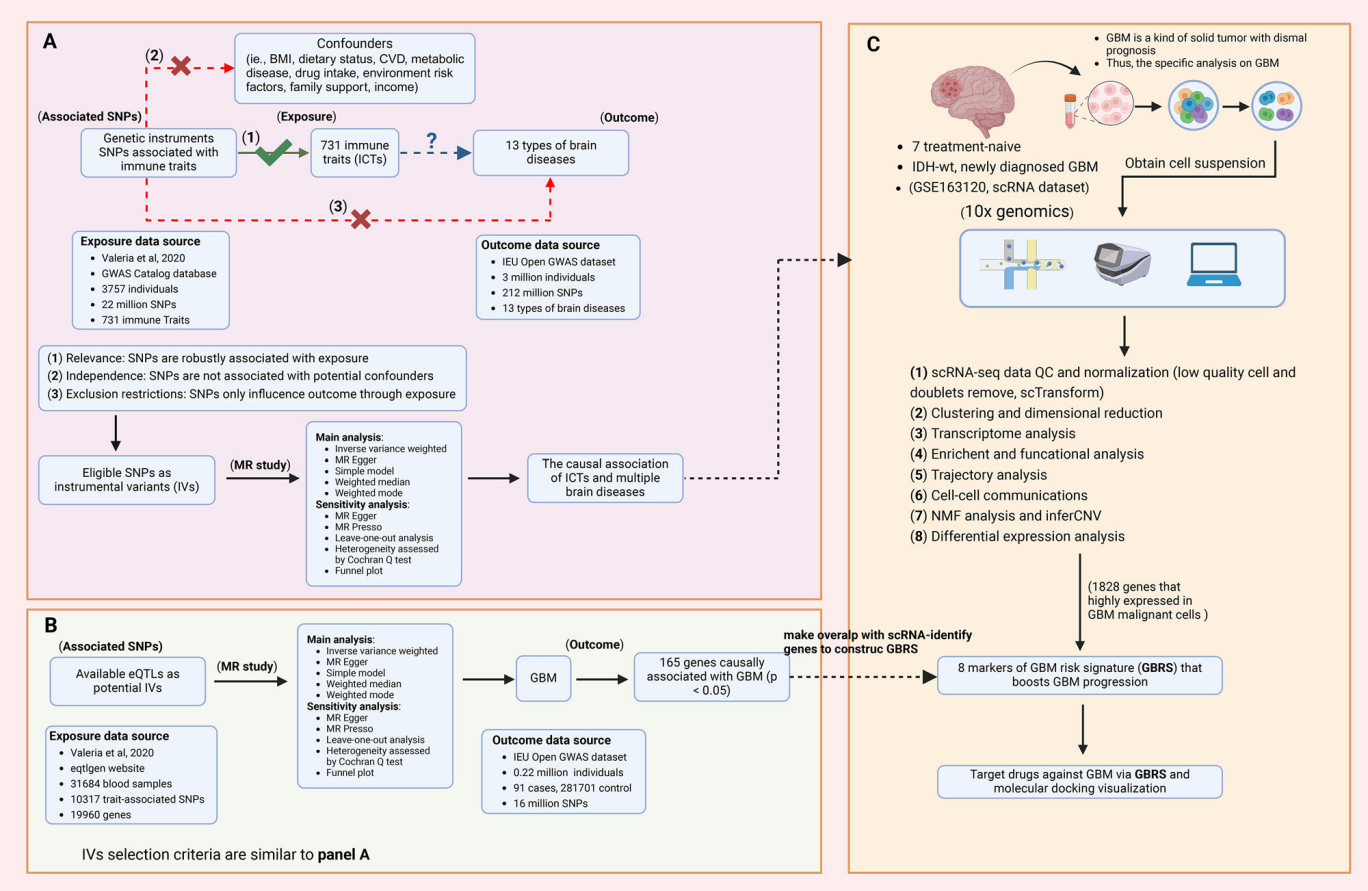
The flowchart delineating the methods employed in both MR and scRNA‐seq analyses within the present investigation. (A) The MR study exploring causal effects of 731 ICTs on 13 brain diseases. (B) The MR study exploring eQTL trait‐associated genes on glioblastoma. (C) The panel showed the inception and features of the scRNA‐seq dataset, the methodologies applied in its analysis, and the framework for establishing the GBRS conducive to glioblastoma progression. BMI, body mass index; CVD, cardiovascular disease; GBM, glioblastoma; GBRS, GBM risk signature. ICT, immune cell trait; IDH, isocitric dehydrogenase; IVs, instrumental variables; MR, Mendelian randomization; NMF, nonnegative matrix factorization; QC, quality control; scRNA‐seq, single‐cell RNA sequencing; SNP, single nucleotide polymorphism; (This figure was created with Biorender [www.biorender.com]).

Genetic variants, established and randomly assigned at conception, serve as unbiased proxies in two‐sample MR to estimate the causal effect of an exposure on an outcome while minimizing confounding and reverse causation. In this study, we investigated the causal relationships between 731 ICTs and a range of brain disorders using two‐sample MR analyses. The MR approach employs genetic variants as instrumental variables (IVs) for risk factors, requiring adherence to three core assumptions for valid causal inference: (1) IVs must be strongly associated with the exposure (relevance assumption); (2) IVs must be independent of confounders affecting the exposure–outcome relationship (independence assumption); and (3) IVs must influence the outcome solely through the exposure, without alternative pathways (Figure 1A).

### 2.2 Exposure and Outcome Data Sources

Summary statistics for 731 ICTs listed in the GWAS Catalog (https://www.ebi.ac.uk/gwas/), spanning GCST90001391 to GCST90002121, were compiled (Orrù et al. 2020). Genome‐wide association study (GWAS) data were derived from 3757 non‐overlapping individuals of European ancestry. A high‐density array, informed by a Sardinian sequence reference panel (Sidore et al. 2015), enabled the estimation of approximately 22 million single‐nucleotide polymorphisms (SNPs), which were tested for associations after adjusting for covariates including age and sex. The 731 ICTs encompassed relative cell counts (RC; *n* = 192), morphologic parameters (MP; *n* = 32), absolute cell counts (AC; *n* = 118), and median fluorescence intensities (MFI; *n* = 389) reflecting cell surface antigen levels (Table S2). Specifically, MP features included panels for conventional dendritic cells (cDCs) and TBNK (T cells, B cells, NK cells), while MFI, RC, and AC features encompassed B cells, cDCs, T cell maturation stages, myeloid cells, monocytes, and TBNK panels.

For outcomes of this MR study, we incorporated 13 distinct types of brain diseases: ischemic stroke (IS), MS, Guillain–Barre syndrome (GBS), PD, amyotrophic lateral sclerosis (ALS), epilepsy, dementia with Lewy bodies (DLB), AD, myasthenia gravis (MG), glioma, GBM, MDD, and schizophrenia in the IEU Open GWAS dataset (https://gwas.mrcieu.ac.uk/) with specific GWAS ID. The selection criteria for GWAS cohorts were as follows: (1) Datasets derived from European populations were prioritized to minimize individual‐level genetic heterogeneity, excluding non‐European datasets or those with unspecified population origins. (2) Datasets were sourced from the IEU Open GWAS platform, ensuring availability and suitability for analysis. (3) Essential metadata, including sample size, number of SNPs, and genome reference, were accessible for each GWAS identifier. (4) Among datasets meeting all criteria, the largest and most recent dataset was selected. Basic information for the outcome results can be found in Table S1. All participants were of European ancestry to minimize genetic heterogeneity.

### 2.3 IV Selection

Genetic variants for ICTs derived from the GWAS database were selected as IVs using a relaxed genome‐wide significance threshold of 1 × 10^−5^ (Vierstra et al. 2020). To ensure independence, SNPs were clumped based on a linkage disequilibrium (LD) threshold of *R* ^2^ < 0.001 within a 10,000 kb window, using the 1000 Genomes Project LD reference panel, retaining the SNP with the lowest *p* value at each locus (Vierstra et al. 2020). The strength of the IVs was evaluated by calculating the *F* statistic, with IVs having an *F* statistic ≤ 10 excluded to mitigate biases from weak instruments. For the 13 brain disorder outcomes, a more stringent threshold was applied, requiring *p* < 5 × 10⁻⁸ and *R* ^2^ < 0.001. To address the untestable exclusion restriction assumption, variants exhibiting potential pleiotropic associations with the 13 brain disorders (*p* < 0.05) were removed, adopting a conservative approach to enhance the reliability of the MR analysis (Hemani et al. 2018; He et al. 2022). The selected IVs for each brain disorder were visualized using Manhattan plots generated with the CMplot package (version 4.5.1).

### 2.4 Statistical Analyses for MR

MR analyses were conducted in accordance with the STROBE‐MR guidelines (Supporting Information) (Skrivankova et al. 2021). Five distinct methods were employed to estimate causal effects: inverse variance weighted (IVW), weighted median, MR‐Egger, weighted mode, and simple mode. The IVW method, which meta‐analyzes Wald‐type ratios of IVs under a multiplicative random‐effects model, and the MR‐Egger method served as the primary approaches for two‐sample MR analyses. Odds ratios (ORs) were reported per standard deviation (SD) increase in the exposure (Bowden et al. 2017). Heterogeneity among IVs was assessed using Cochran's *Q* test. To address the no measurement error (NOME) assumption underlying IVW estimates, MR pleiotropy residual sum and outlier (MR‐PRESSO) and MR‐Egger methods were utilized to detect and adjust for potential horizontal pleiotropy. The MR‐PRESSO global test evaluates overall horizontal pleiotropy by comparing the observed residual sum of squares for all variants to the expected distance under the null hypothesis of no pleiotropy (Verbanck et al. 2018). Conversely, the MR‐Egger method employs weighted regression to estimate an intercept, indicating directional pleiotropy when significantly nonzero (*p* < 0.05) (Bowden et al. 2015). Causal effects were considered nominally significant (suggestive) when the IVW *p* < 0.05 and sensitivity analyses revealed no contradictory findings. Leave‐one‐out (LOO) analyses were performed to identify IV outliers substantially influencing causal estimates (Burgess et al. 2017). Funnel and scatter plots were generated to visually inspect symmetry and effect estimates. All MR analyses were conducted and visualized using R (version 4.3.0) with the packages TwoSampleMR (version 0.5.8), ieugwasr (version 0.1.5), VariantAnnotation (version 1.44.1), gwasglue (version 0.0.0.9), and forestploter (version 1.0.0).

### 2.5 Colocalization Analysis

Colocalization analysis was employed to evaluate shared causal variants between traits within genomic regions. Initially, nominally significant causal associations identified by the IVW method were selected and adjusted for multiple testing using the Benjamini–Hochberg (BH) procedure. IVW results with a false discovery rate (FDR) < 0.05 were deemed significant and advanced to colocalization analysis (Aggarwal et al. 2022). Then, the significant results were prepared for colocalization analysis. A Bayesian approach, implemented using the “coloc” R package (version 5.2.3), was used to estimate the posterior probability of shared genetic effects, thereby enhancing the precision of our findings (Giambartolomei et al. 2014). A posterior probability for the hypothesis of a shared causal variant (PP.H4) exceeding 0.80 was considered indicative of robust colocalization between traits and associations with multiple brain disorders. Such strong colocalization evidence suggests that the observed genetic associations may be driven by shared biological mechanisms.

### 2.6 scRNA‐seq Analysis on GBM

MR analyses identified potential immune cell‐related risk and protective factors for a range of neurological disorders, establishing causal associations; however, the underlying biological mechanisms remained elusive. To address this, scRNA‐seq data were integrated to cross‐validate MR findings, with a particular focus on GBM, thereby enhancing multi‐omics evidence beyond GWAS data alone. Advanced scRNA‐seq algorithms enabled detailed characterization of immune cell niches and communication pathways within the TME, aligning with the objectives of the MR analyses (Lotfollahi et al. 2024). GBM, the most prevalent primary malignant brain tumor, is characterized by limited treatment efficacy and a highly aggressive phenotype, distinguishing it from other neurological disorders (Weller et al. 2024). Despite standard‐of‐care interventions, the median overall survival (OS) for GBM patients remains approximately 20 months (Stupp et al. 2005). Consequently, the identification of reliable biomarkers for GBM diagnosis, early intervention, and treatment is critical. The integration of MR and scRNA‐seq analyses facilitated the discovery of robust biomarkers and provided insights into GBM's intratumoral heterogeneity and comprehensive TME features. Notably, while MR analyses of neurological disorders relied on blood‐derived samples, such data alone are insufficient to fully elucidate the biological trajectories of malignant tumors like GBM, and the resulting MR estimates may lack precision for clinical applications. Analyzing both blood and tumor samples offers greater validity and more comprehensive evidence. These multi‐omics findings may establish a framework for future GBM research, advancing the understanding of its complex biology.

### 2.7 Integration, Dimensionality Reduction, Clustering, and Cell Annotation for scRNA‐seq Data

scRNA‐seq data from seven treatment‐naïve, isocitrate dehydrogenase wild‐type (IDH‐wt), newly diagnosed GBM patients were obtained from the GSE163120 dataset and analyzed. Detailed protocols for tissue acquisition, library preparation, sequencing, and preprocessing have been previously described (Pombo Antunes et al. 2021). Low‐quality cells were excluded based on elevated mitochondrial, ribosomal, or hemoglobin gene expression ratios. Expression matrices from all samples were converted into a Seurat object using the Seurat package (version 4.3.0). To address potential doublet contamination, the scDblFinder package (version 1.15.4) was applied, assuming a 4% doublet proportion and incorporating gene count and unique molecular identifier (UMI) metrics for cell filtering (Germain et al. 2022). Data normalization was performed using Regularized Negative Binomial Regression (RNBR) via the SCTransform function (version 0.3.5) in Seurat, identifying 3000 highly variable genes (HVGs) for subsequent principal component analysis (PCA) (Hafemeister and Satija 2019). Batch effects across samples were corrected using Harmony (version 0.1.1), which generated an integrated PCA embedding for dimensionality reduction (Korsunsky et al. 2019). The top 30 principal components (PCs) were selected and used for clustering with the “FindNeighbors” and “FindClusters” functions in Seurat, applying resolutions ranging from 0.5 to 1.5. Clusters were projected onto a two‐dimensional uniform manifold approximation and projection (UMAP) space. Marker genes for each cluster were identified using the “FindAllMarkers” function in Seurat, and cell types were annotated by combining the SingleR package with established cell marker genes from the literature.

### 2.8 Determination of Malignant Cells

To identify malignant cells, copy number variation (CNV) signatures were inferred using the inferCNV package (version 1.5.0., https://github.com/broadinstitute/inferCNV) with default parameters. Immune cell clusters were used as a reference to distinguish malignant cells. To validate these inferences, CNV scores were calculated for putative malignant cells, with higher scores indicating greater copy number alterations within a cell cluster.

### 2.9 GBM Risk Signature Construction

To comprehensively leverage expression eQTL data, both cis‐ and trans‐eQTL datasets were obtained from the eQTLGen Consortium (https://www.eqtlgen.org/). This dataset included 31,684 blood samples comprising 10,317 trait‐associated SNPs and 19,960 genes (Võsa et al. 2021). IVs were selected from these trait‐associated SNPs, with trait‐associated genes as the exposure and GBM as the outcome. IV selection and MR analyses followed previously described methods, applying a genome‐wide significance threshold of *p* < 5 × 10⁻⁸ for IVs. Using eQTL data, genes causally linked to GBM development and aggressiveness were identified (Figure 1B). To develop a refined glioblastoma risk signature (GBRS) for predicting tumor progression, differentially expressed genes (DEGs) between malignant cells and other clusters were identified using the “FindAllMarkers” function in the Seurat package, with thresholds of |log₂ fold change| > 0.25 and minimum expression percentage > 0.25. DEGs highly expressed in malignant cells were cross‐referenced with eQTL results to select markers for GBRS construction (Figure 1C). The resulting GBRS comprised eight markers, and the “AddModuleScore” function in Seurat was used to calculate GBRS scores and characterize its features.

### 2.10 Nonnegative Matrix Factorization Analysis of the Sample Heterogeneity Expression Signatures

To characterize the dominant expression signatures and cellular states of malignant cells in scRNA‐seq samples, nonnegative matrix factorization (NMF) was performed as previously described (Hovestadt et al. 2019). For seven samples, NMF was applied using a factorization rank of 60 and 3000 HVGs, generating distinct NMF programs for each iteration. All negative values in the scaled, normalized expression matrix were set to zero. To investigate shared features among malignant cells, the R AUCell package was used to calculate scores for each NMF program, followed by hierarchical clustering of scores and computation of Pearson's correlation coefficients. This analysis identified six NMF metaprograms across malignant cells. To define malignant cell states, the “AddModuleScore” function in the Seurat package was employed to score these six NMF metaprograms using the top 25 marker genes (metaGenes) associated with each metaprogram.

### 2.11 Gene Ontology and Pathway Enrichment Analysis

Functional enrichment analysis was conducted to investigate gene ontology (GO), Hallmark, and Kyoto Encyclopedia of Genes and Genomes (KEGG) pathways. GO enrichment analysis of DEGs was performed using the clusterProfiler package, with gene sets obtained from the MSigDB database (Yu et al. 2012). To assess pathway activity across 14 predefined signaling pathways (androgen, estrogen, EGFR, hypoxia, JAK‐STAT, MAPK, NFκB, PI3K, p53, TGFβ, TNFα, TRAIL, VEGF, and WNT), the PROGENy framework was applied in combination with decoupleR algorithms (Schubert et al. 2018; Garcia‐Alonso et al. 2019). The normalized expression matrix was used as input for PROGENy, generating a Seurat object with independent PROGENy scores for each cell.

### 2.12 CytoTRACE and Pseudotime Analysis

Developmental trajectories of malignant cells and cells corresponding to NMF‐defined metaprograms were inferred using Monocle2 (v2.28.0) (Qiu et al. 2017). Briefly, normalized gene expression matrices were exported from Seurat and subsequently imported into Monocle2 to construct a CellDataSet object. Cells were then ordered along a pseudotime trajectory based on variable genes identified by the differentialGeneTest function (*q* < 0.001). Dimensionality reduction was performed using the reduceDimension function (employing the “DDRTree” algorithm and norm_method = “none” parameter) to reconstruct cellular differentiation pathways. Genes exhibiting dynamic expression patterns along the inferred pseudotime trajectory were visualized using the plot_pseudotime_heatmap function. The plot_cell_trajectory function was utilized to visualize the cell trajectories for distinct cell populations.

Complementarily, cellular differentiation potential was assessed using the CytoTRACE algorithm (v0.3.3) (Gulati et al. 2020). CytoTRACE analyzes single‐cell transcriptomic data to predict differentiation states, assigning each cell a score ranging from 0 to 1. Higher scores are indicative of a less differentiated (i.e., more stem‐like) state, whereas lower scores denote a more differentiated phenotype.

### 2.13 Cell–Cell Interaction

To elucidate potential ligand–receptor (L–R) interactions between distinct cell populations, we utilized the LIANA framework (v0.1.13). LIANA integrates results from multiple algorithms; specifically, we employed its “natmi,” “connectome,” “logfc,” “sca,” and “cellphonedb” methods. Interactions were considered significant if their aggregated *p* ≤ 0.05 (Dimitrov et al. 2022). Subsequently, the CellChat package (v1.6.1) was used for a more comprehensive analysis and visualization of intercellular communication networks. This analysis focused on interactions categorized within CellChat's curated databases, including those mediated by “secreted signaling,” “extracellular matrix (ECM)‐receptor” interactions, and direct “cell–cell contact” mechanisms (Jin et al. 2021).

### 2.14 Drug Prediction and Molecular Docking

Predicting protein–drug interactions plays a pivotal role in comprehending the structural characteristics essential for drug sensitivity. GBRS was submitted to the Drug Signatures Database (DSigDB, https://dsigdb.tanlab.org/DSigDBv1.0/) containing 22,527 gene sets relevant to drug prediction. Visualization was finalized with the Enrichr platform (https://maayanlab.cloud/Enrichr/), and candidate drugs were ranked in the ascending order of their adjusted *p* values. An adjusted *p* < 0.05 was considered statistically significant. Small molecules and the hub targets were docked using AutoDock Vina (Scripps Research, San Diego, CA). These results of docking were evaluated and analyzed using the PLIP system (https://plip‐tool.biotec.tu‐dresden.de/plip‐web/plip/index). The molecular docking (MD) outcomes of the two‐dimensional structures were visualized using the LIGPLOT software version 4.5.3 (European Bioinformatics Institute, Cambridge, UK), while three‐dimensional structures were generated using PyMOL (https://pymol.org/). Protein structures were obtained from PDB (https://www.pdb.org/) or AlphaFold (https://alphafold.com/); data on selected compounds was retrieved from PubChem (https://pubchem.ncbi.nlm.nih.gov/), and binding energy calculations were performed using Chem3D Pro software version 9.0 (PerkinElmer, USA).

### 2.15 Ethics Approval

Each study included in the MR and scRNA‐seq data underwent approval by local research ethics committees or Institutional Review Boards, and all participants provided their informed consent.

## 3 Results

### 3.1 IVs Selection and MR Results

**FIGURE 2:**
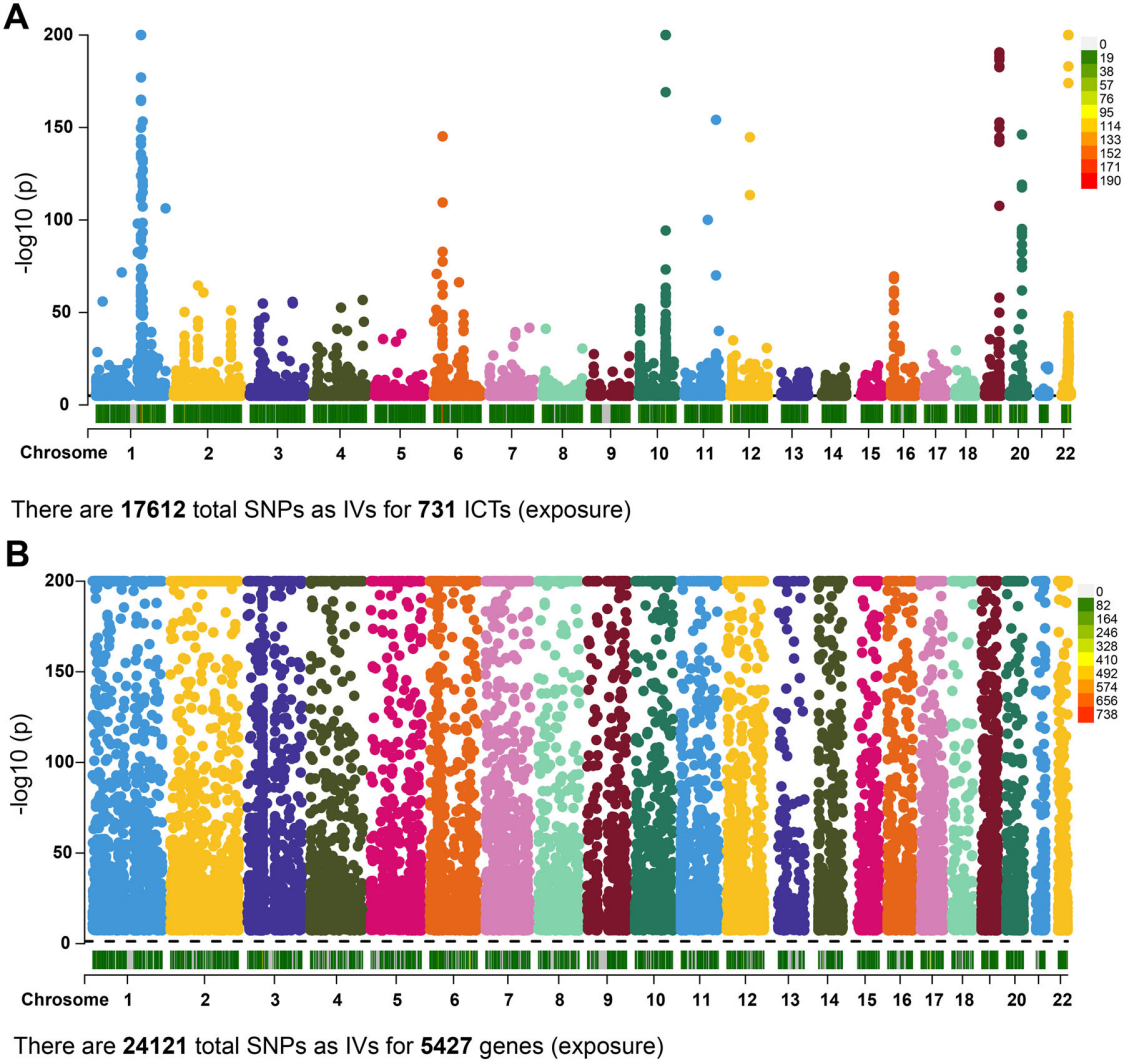
The Manhattan plots delineating used IVs in this MR study. (A) The IVs employed in the MR investigation explored the impact of 731 ICTs on 13 brain diseases. (B) The IVs utilized in the MR analysis probed the association of eQTL‐linked genes with glioblastoma. ICT, immune cell trait; IVs, instrumental variables.

The study design, encompassing MR analyses of ICTs and eQTL exposures, as well as scRNA‐seq analyses, was illustrated in Figure 1A–C. A total of 17,612 SNPs were identified as IVs for 731 ICTs (Figure 2A). Additionally, 24,121 IVs were selected for 5427 eQTL‐paired genes (Figure 2B).

**FIGURE 3:**
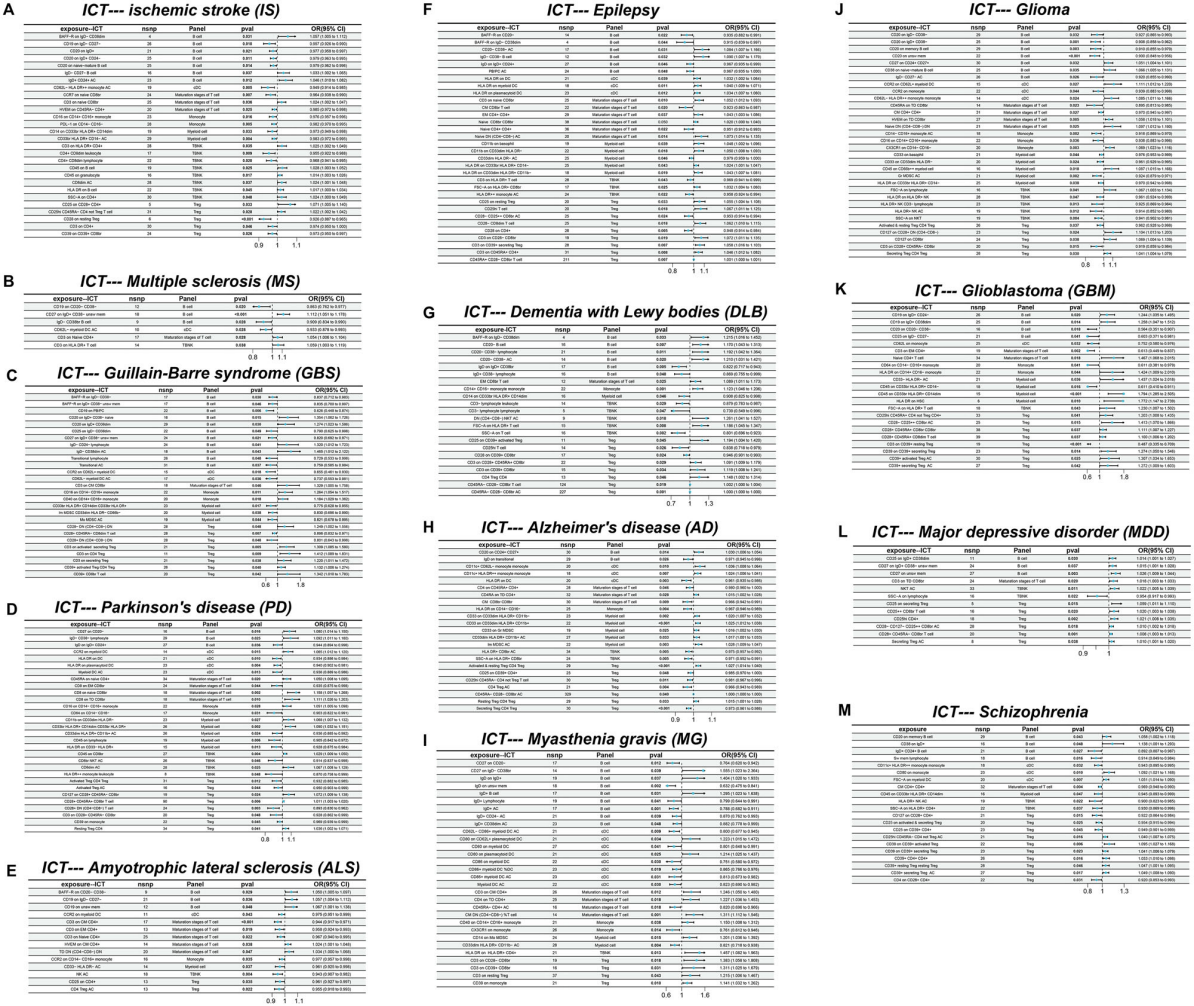
Forest plots elucidating MR outcomes concerning the impact of 731 ICTs on 13 brain diseases. Each specific result pertaining to the 13 types of brain diseases was depicted in Panels A–M, with individual subtitles atop each panel delineating the detailed associations. Further elucidation regarding the meanings and descriptions of each ICT can be found in Table S2 and the study of Orrù et al. 2020. An OR and 95% CI greater than 1 signify a causal association of the ICT with an increased risk for the outcome, while an OR and 95% CI less than 1 indicate an association with a decreased risk for the outcome. A *p* value less than 0.05 denotes statistically significant findings. 95% CI, 95% confidence interval; ICTs, immune cell traits; MR, Mendelian randomization; nsnp, number of single nucleotide polymorphism; OR, odd ratio.

The causal relationships between 731 ICTs and 13 brain diseases were characterized through MR analyses (Figure 3; Tables S3–S15). Key findings include the following: (1) Individual ICTs exhibited divergent roles across brain diseases, conferring either protective or risk‐enhancing effects; (2) In nonmalignant neurological and psychiatric disorders, including MS, PD, ALS, DLB, AD, and epilepsy, lymphocyte traits were suggestive of being consistently associated with increased susceptibility, highlighting their critical role in the disease microenvironment; (3) myeloid cell traits displayed inconsistent associations, with positive correlations for AD and epilepsy risk but negative correlations for IS and GBS; (4) and malignant disorders, such as glioma and GBM, exhibited distinct immune profiles. B cells, cDCs, and TBNK traits were nominally associated with reduced susceptibility, whereas myeloid cell and Tregs traits were linked to increased risk. These patterns underscore the contrasting immune microenvironments of malignant versus nonmalignant brain diseases. Notably, two ICTs demonstrated potential protective effects against GBM: CD33bright HLA‐DR^+^ CD14^−^ myeloid cells and CD39^+^ resting Tregs. Unlike unconventional Tregs (*LAG3* ^+^ *FOXP3* ^−^) and activated Tregs, which express elevated levels of checkpoint receptors (*TNFRSF4*, *TNFRSF18*), immune response activation genes (*DUSP2*, *DUSP4*), and NF‐κB signaling genes (*NFKBIA*, *TNFAIP3*, *NFKBIZ*), resting Tregs upregulate stress response genes (*HSPA1A*, *HSPA1B*) (Lang and Raffi 2019; Spasevska et al. 2023).

It is widely acknowledged that CD33^bright^ and HLA‐DR^−^ are the hallmarks of myeloid‐derived suppressor cells (MDSCs), which exhibit obvious anti‐inflammatory function and immunosuppressive properties. In this context, this CD33^bright^ HLA‐DR^+^ and CD14^−^ trait could be opposite to MDSCs, suggesting proinflammatory status (Kumar et al. 2016; Horzum et al. 2021). For MR analysis on the CD33^bright^ HLA‐DR^+^ CD14^−^ myeloid cell trait, all five MR methods yielded consistent results, of which the OR was 0.61 (95% confidence interval [95% CI], 0.41–0.91; *p *= 0.016) for the IVW method and 0.18 (95% CI, 0.05–0.68; *p *= 0.022) for the MR‐Egger method (Figure S1). Regarding the CD39^+^ resting Tregs trait, five MR methods showed consistent results. Notably, the IVW method illustrated the OR was 0.49 (95% CI, 0.34–0.71; *p *< 0.001), and the MR‐Egger method displayed the OR was 0.47 (95% CI, 0.26–0.85; *p *= 0.022) (Figure S2). Then, MR‐PRESSO detected no evident horizontal pleiotropy (*p *= 0.662, *p *= 0.309, respectively), and MR‐Egger regression intercepts exhibited no significant findings (*p *= 0.078, *p *= 0.893, respectively). The LOO test did not reveal any IVs disproportionately influencing the final estimates. Cochran *Q* tests did not provide evidence for significant heterogeneity (*p *= 0.814 [MR‐Egger], *p *= 0.633 [IVW]; *p *= 0.201 [MR‐Egger], *p *= 0.250 [IVW], respectively).

For the significant causal associations with FDR < 0.05, rs2949661 and rs9916257 associated with CD247 and SLFN12L impacted CD3 on CM CD4^+^ and NK AC leading to a decreased risk of ALS; CD33 could influence different CD33^+^ ICTs to enhance the risk of AD under rs3865444 and rs2455069, while rs3087456‐CIITA could reduce AD risk by impacting HLA DR^+^ CD8br AC. For GBM, rs1723016/rs2995089‐CD247 influenced CD3 on CD39^+^ resting Treg and CD3 on EM CD4^+^ to significantly reduce the risk; CD27 on unswitched memory increased the MDD risk through colocalized rs4810485‐CD40 biologically (Table 1).

**TABLE 1:** The colocalization results on causal associations with FDR < 0.05.

SNP ID	Chr	Pos	Effect allele	Other allele	Exposure						Outcome						Adjusted MR results			Colocalizaion analysis result	
					Exposure	Beta.exposure	EAF.exposure	*p*val.exposure	Se.exposure	Samplesize	Outcome	Beta.outcome	EAF.outcome	Se.outcome	*p*val.outcome	Samplesize	OR(95% CI)	*p*val	FDR^a^	PP.H4	Gene/marker
rs2949661	1	167424924	T	C	CD3 on CM CD4^+^	0.567	0.4463	3.45E‐99	0.0258	2912	ALS	−0.05	NA	0.0114	1.14E‐05	138086	0.94 (0.92–0.97)	7.29E‐05	0.004	1	CD247
rs9916257	17	33797371	T	G	NK AC	−0.2005	0.5686	8.82E‐17	0.02398	3653	ALS	0.0098	NA	0.0109	0.3702	138086	0.94 (0.91–0.98)	0.004	0.043	1	SLFN12L
rs3865444	19	51727962	A	C	CD33 on CD14^+^ monocyte	−1.143	0.2121	1.05E‐190	0.03378	1634	AD	−0.0337	0.3114	0.0093	0.000309999	85934	1.03 (1.01–1.04)	7.85E‐05	0.004	1	CD33
rs3865444	19	51727962	A	C	CD33 on CD33dim HLA DR^+^ CD11b^−^	−1.119	0.2121	2.40E‐183	0.03392	1634	AD	−0.0337	0.3114	0.0093	0.000309999	85934	1.03 (1.01–1.04)	7.85E‐05	0.004	1	CD33
rs3865444	19	51727962	A	C	CD33 on CD33dim HLA DR^+^ CD11b^+^	−1.139	0.2122	2.38E‐191	0.03358	1633	AD	−0.0337	0.3114	0.0093	0.000309999	85934	1.02 (1.01–1.04)	1.00E‐04	0.004	1	CD33
rs3865444	19	51727962	A	C	CD33 on CD66b^++^ myeloid cell	−0.9497	0.2094	2.39E‐108	0.0394	1464	AD	−0.0337	0.3114	0.0093	0.000309999	85934	1.02 (1.01–1.04)	0.002	0.022	1	CD33
rs12778618	10	95633120	C	T	CD39^+^ CD8br AC	0.8528	0.0461	3.23E‐42	0.06178	3408	AD	0.0413	0.016	0.0347	0.234	85934	0.98 (0.96–0.99)	0.007	0.038	1	NA
rs34039593	6	32583099	G	T	HLA DR on DC	1.075	0.1174	3.67E‐110	0.04613	2871	AD	−0.0975	0.1613	0.0114	1.05E‐17	85934	0.96 (0.94–0.99)	0.003	0.024	1	NA
rs2455069	19	51728641	G	A	Im MDSC AC	0.3938	0.5197	1.25E‐32	0.03247	1857	AD	0.0377	0.423	0.0088	1.91E‐05	85934	1.03 (1.01–1.05)	0.003	0.024	1	CD33
rs34291045	6	32566398	T	A	HLA DR on CD14^−^ CD16^−^	0.8124	0.1171	1.53E‐83	0.04087	3629	AD	−0.0929	0.1625	0.0112	1.38E‐16	85934	0.97 (0.95–0.99)	0.004	0.027	1	NA
rs71632979	1	161526397	G	A	Myeloid DC %DC	0.6258	0.2447	8.46E‐99	0.0287	3410	AD	0.0243	0.1289	0.0124	0.0493197	85934	1.03 (1.01–1.06)	0.005	0.031	1	NA
rs3087456	16	10970902	A	G	HLA DR^+^ CD8br AC	−0.4286	0.766	5.57E‐52	0.0278	3579	AD	0.0164	0.7262	0.0095	0.0822906	85934	0.98 (0.96–0.99)	0.007	0.038	1	CIITA
rs1723016	1	167433056	C	T	CD3 on CD39^+^ resting Treg	0.3265	0.4579	1.57E‐31	0.02759	2649	GBM	−0.2691	0.2691	0.1664	0.1058	NA	0.49 (0.34–0.71)	2.00E‐04	0.019	1	CD247
rs2995089	1	167431193	A	G	CD3 on EM CD4^+^	0.4829	0.4461	2.05E‐71	0.02629	2912	GBM	−0.2264	0.2593	0.1684	0.1787	NA	0.61 (0.45–0.84)	0.002	0.046	1	CD247
																					
rs4810485	20	44747947	G	T	CD27 on unsw mem	−0.4783	0.6313	5.93E‐78	0.02498	3656	MDD	0.6313	0.755294	0.0151147	0.0374024	92957	1.03 (1.01–1.04)	0.003	0.038	1	CD40
rs34933238	5	23346191	G	A	CD28^+^ CD45RA^−^ CD8br %T cell	0.374	0.0339	5.24E‐08	0.06857	3440	MDD	−0.0139135	0.107926	0.0150752	0.356041	92957	1.01 (1.00–1.01)	0.001	0.038	1	NA

^a^ FDR is adjusted p value based on the Benjamini–Hochberg method.

Abbreviations: 95% CI, 95% confidence interval; CM, central memory; DC, dendritic cell; EAF, effect allele frequency; EM, effector memory; FDR, false discovery rate; HLA, human leukocyte antigen; MDSC, myeloid‐derived suppressor cell; MR, Mendelian randomization; NA, not available; NK, natural killer cell; OR, odd ratio; Tregs, regulatory T cells.

### 3.2 Single‐Cell Transcriptomic Atlas and Cell Typing in GBM

**FIGURE 4:**
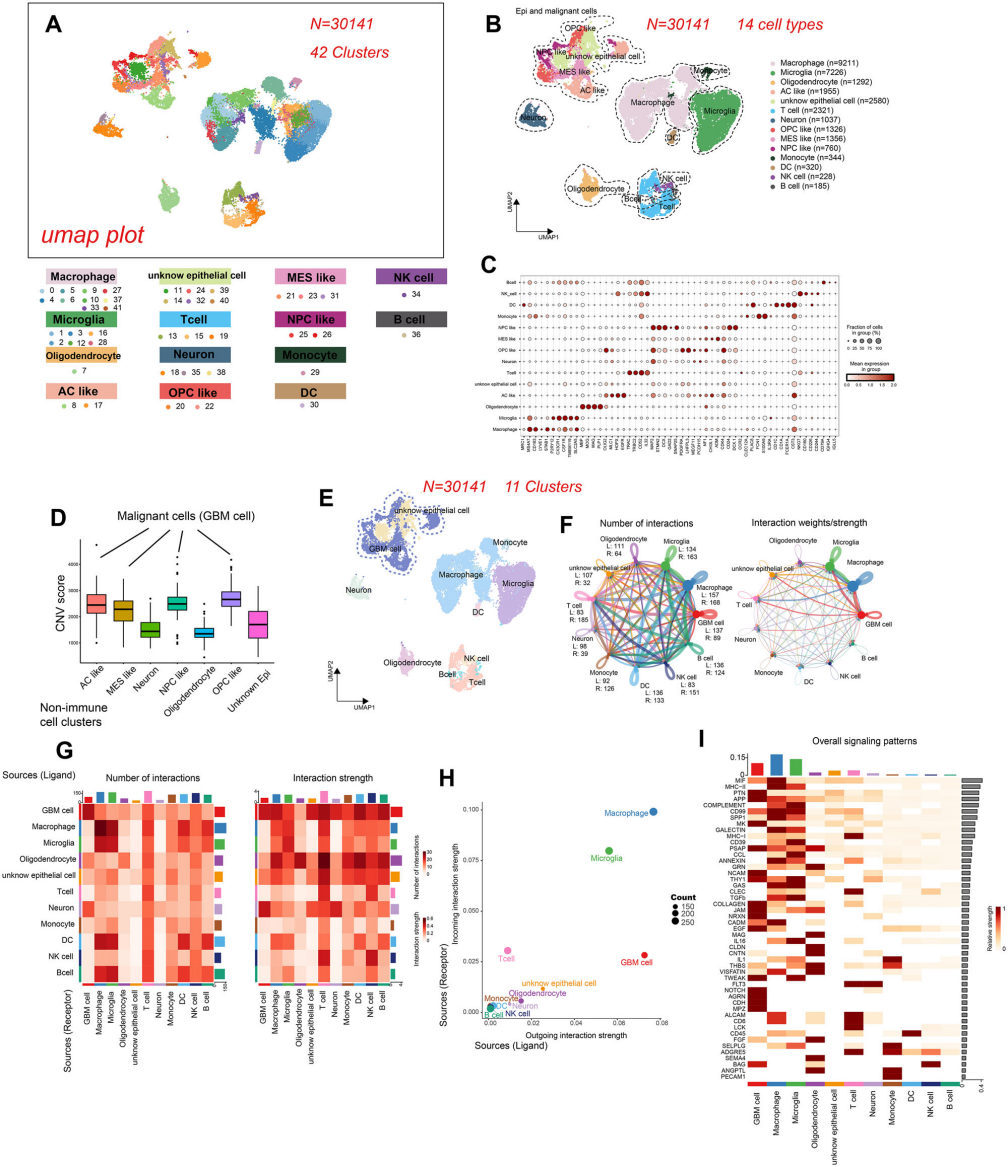
scRNA‐seq identifies cell clusters in GBM and cell–cell interactions among 11 available cell types. (A) UMAP representation and initial cluster assignment for integrated scRNA‐seq samples of GBM, comprising 30,141 cells distributed across 42 clusters. (B) UMAP representation further refines the clustering, integrating the 42 clusters into 14 annotated cell types based on previous evidence and biological knowledge. Cell type alignments are indicated in different colors. (C) The dot plot displayed representative marker genes for each cell type, with dot size proportional to the fraction of cells expressing specific genes and color intensity corresponding to the relative expression of those genes. (D) Boxplot displayed CNV score inferred by inferCNV algorithm among non‐immune cells. AC‐like, MES‐like, NPC‐like, and OPC‐like clusters demonstrating notably higher CNV scores, indicative of more CNV events, were subsequently selected as malignant cells (GBM cells) for further analysis. (E) By integrating AC‐like, MES‐like, NPC‐like, and OPC‐like clusters into the GBM cells, the UMAP plot illustrated the location and distribution for each cluster. (F) The number and strength of interactions across the 11 identified clusters, with arrows representing interactions from ligands (L) to receptors (R) and line width indicating the number or strength of interactions, where bolder lines denote more interactions or stronger interactions. (G) The heatmap revealed the number and strength of interactions between clusters, with deeper red indicating higher numbers or strengths of interactions. Clusters marked on the *X*‐axis were Ls, and on the *Y*‐axis were Rs. (H) The dot plot showed the overall incoming and outgoing strengths for each cluster, with clusters closer to the top‐right corner exhibiting activity in both incoming and outgoing signaling patterns. (I) The heatmap showcased overall signaling patterns across the 11 cell types, with the bar on the top indicating the overall strength across presented pathways in each cell type and the grey bar on the right illustrating the overall strength across the 11 cell types in each pathway, where deeper red signifies higher relative strength. AC‐like, astrocyte‐like; CNV, copy number variation; DC, dendritic cell; MES‐like, mesenchymal‐like; NK cell, natural killer cell; NPC‐like, neural‐progenitor‐like; OPC‐like, neural‐progenitor‐like; UMAP, uniform manifold approximation and projection.

Following rigorous quality control procedures, we obtained a total of 30,141 cells from all samples for subsequent analysis. These cells were categorized into 42 clusters (resolution: 1.5) (Figure 4A) representing 12 distinct cell types incorporating macrophage (*n* = 9211), microglia (*n* = 7226), oligodendrocyte (*n* = 1292), astrocyte‐like (AC‐like) (*n* = 1955), neural‐progenitor‐like (OPC‐like) (*n* = 1326), mesenchymal‐like (MES‐like) (*n* = 1356), neural‐progenitor‐like (NPC‐like) (*n* = 760) cell, T cell (*n* = 2321), neuron (*n* = 1037), monocyte (*n* = 344), DC (*n* = 320), NK cell (*n* = 228), B cell (*n* = 185), and unknown epithelial cell (*n* = 2580) (Figure 4B). These cell types were annotated using established marker genes (Figure 4C), and the identified cell types associated with GBM were consistent with findings from previous studies (Neftel et al. 2019; Patel et al. 2014). Using immune cells as references, we inferred the CNV status for potential malignant cells and other cell types. Notably, OPC‐like, NPC‐like, AC‐like, and MES‐like cells exhibited significantly higher CNV scores compared to neurons and oligodendrocytes, and slightly higher than unknown epithelial cells, suggesting a greater frequency of CNV events (Figure 4D and Figure S3). Consequently, it became apparent that while not all, certain unknown epithelial cells harbored malignancy. To ensure the utmost accuracy, AC‐like, NPC‐like, OPC‐like, and MES‐like cells were integrated into the GBM cell population, representing the malignant cells (Figure 4E). Moreover, we visualized cell–cell interactions across GBM and other cells using CellChat (Figure 4F), incorporating datasets related to secreted signaling, ECM‐receptor, and cell–cell contact (Table S16). Remarkably, macrophages exhibited the highest number of ligands (L), followed by GBM cells, microglia, and DCs, whereas T cells displayed the highest number of receptors (R) (Figure 4F,G). Regarding interaction strength, macrophages exerted the most robust influence on both L and R, with microglia ranking second in R strength and GBM cells second in L strength (Figure 4F,G,H). Analyzing the involved pathways, *MIF*, *MHC‐II*, *PTN*, *APP*, and *COMPLEMENT* emerged as the top five active pathways, predominantly enriched in macrophages, microglia, and GBM cells (Figure 4I). To elucidate the specific communication patterns between GBM cells and macrophages, as well as microglia, we employed the LIANA algorithm. This approach offers several advantages, including overcoming potential biases and imbalances inherent in certain methods; for example, CellPhoneDB is likely to report signaling pathways related to inflammation and proliferation and provide more robust and objective L–R pairs by integrating data from five methods and generating aggregate rank values (Dimitrov et al. 2022) (Table S17). Notably, the top five L–R pairs based on aggregate rank from GBM cell to macrophage were *APP‐CD74*, *SPAPC‐ENG*, *MDK‐SORL1*, *MDK‐LRP1*, and *VEGFA‐ITGB1*, and to microglia were *MDK‐SORL1*, GAL‐*HLA‐DPA1*, *THY‐ITGAX_ITGB2*, *THY1‐ITGAM_ITGB2*, and *GNAS‐ADRB2* (Table S17).

### 3.3 Characterization of NMF Subtypes and Heterogeneity of GBM Cell

**FIGURE 5:**
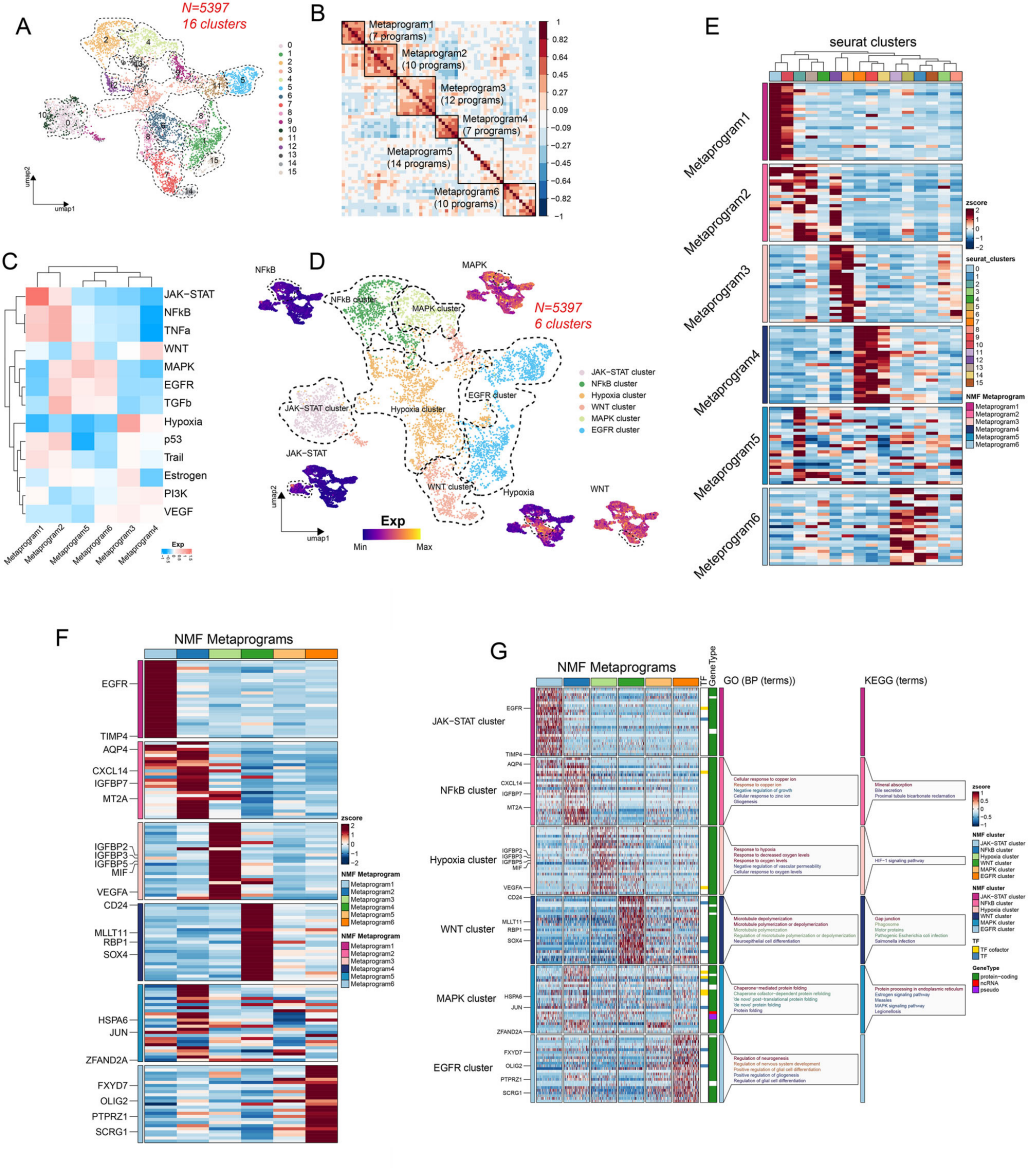
scRNA‐seq identifies gene expression programs driving cell states in GBM cells. (A) UMAP aligned 5397 GBM cells into 16 clusters with different colors. (B) Employing NMF and consensus clustering, 60 identified programs could be classified into six metaprograms, with Pearson's correlation relevance depicted on the bar to the right. (C) The PROGENy heatmap indicated the enrichment of 13 pathways in six metaprograms. (D) UMAP representation, distribution, and cell states were identified for six metaprograms. From the UMAP plot, we noticed that 5397 GBM cells were identified into six cell states of the JAK‐STAT cluster, NFκB cluster, hypoxia cluster, WNT cluster, MAPK cluster, and EGFR cluster. (E–F) The average expression of metaGene for each metaprogram in heatmaps. MetaGene average expression in 16 UMAP clusters (E) and in six metaprograms (F). The expression was scaled and visualized in *Z*‐score. (G) The meraGene expression alongside some representative markers in six cell states was displayed in the heatmap. Representative markers were annotated on the left of the heatmap, and GO and KEGG results for each cell state were provided on the right. GO and KEGG function results were also summarized in Table S20. The expression was scaled and visualized in *Z*‐score. GO, gene ontology; KEGG, the Kyoto Encyclopedia of Genes and Genomes.

Based on 5397 GBM cells, we identified 16 clusters via UMAP (resolution: 1.0) (Figure 5A). To comprehensively characterize the intratumoral heterogeneity of malignant cells, we conducted an analysis of coexpressed gene clusters within each patient using NMF and obtained 60 programs. Subsequently, we identified six prominent metaprograms through Pearson's correlation analysis and consensus clustering (Figure 5B). From representative genes in each metaprogram (Table S18), we identified the top 25 as metaGene, representing each metaprogram (Table S19). The heatmap displayed the metaGene expression in 16 Seurat clusters (Figure 5E), facilitating the identification of clusters within the same metaprogram and enabling further classification of GBM at the single‐cell level. Utilizing PROGENy, we observed enrichment of Metaprogram 1 in the *JAK‐STAT* pathway, Metaprogram 2 in the *NFκB* and *TNF‐α* pathways, Metaprogram 3 in the hypoxia pathway, Metaprogram 4 in the WNT pathway, Metaprogram 5 in the *MAPK* and *WNT* pathways, and Metaprogram 6 in the *MAPK* and *EGFR* pathways (Figure 5C). To elucidate the cell state and molecular function of each metaprogram, we plotted the metaGene expression for each metaprogram in a heatmap and annotated some key metaGenes (Figure 5F), and the six metaprograms could be aligned into “JAK‐STAT cluster,” “NFκB cluster,” “hypoxia cluster,” “WNT cluster,” “MAPK cluster,” and “EGFR cluster” in UMAP (Figure 5D). Additionally, we identified genes such as *EGFR* and *TIMP4* that were highly expressed in the JAK‐STAT cluster; *AQP4*, *CXCL14*, and *IGFBP7* in the NFκB cluster; *VEGFA* and *IGFBP2*/*3* in the hypoxia cluster; *RBP1* and *SOX4* in the WNT cluster; stress response genes *HSPA6* and *JUN* in the MAPK cluster; and malignant‐related gene *PTPRZ1* and oligodendrocyte marker *OLIG2* in the EGFR cluster (Figure 5G). Based on available GO and KEGG results derived from metaGenes for each aligned GBM cluster, the hypoxia cluster was primarily associated with hypoxia and oxygen level, related to the HIF‐1 signaling pathway; the NFκB cluster was closely linked to mineral metabolism and gliogenesis; the MAPK cluster was associated with chaperone‐mediated protein processing, while the EGFR cluster regulated gliogenesis, neurogenesis, and glial cell differentiation (Figure 5G, Figure S4B–F). Regarding the JAK‐STAT cluster, while fewer profound GO and KEGG terms were identified, it might be associated with phospholipase activity (Table S20, Figure S4A).

### 3.4 Construction and Characterization of the GBRS

**FIGURE 6:**
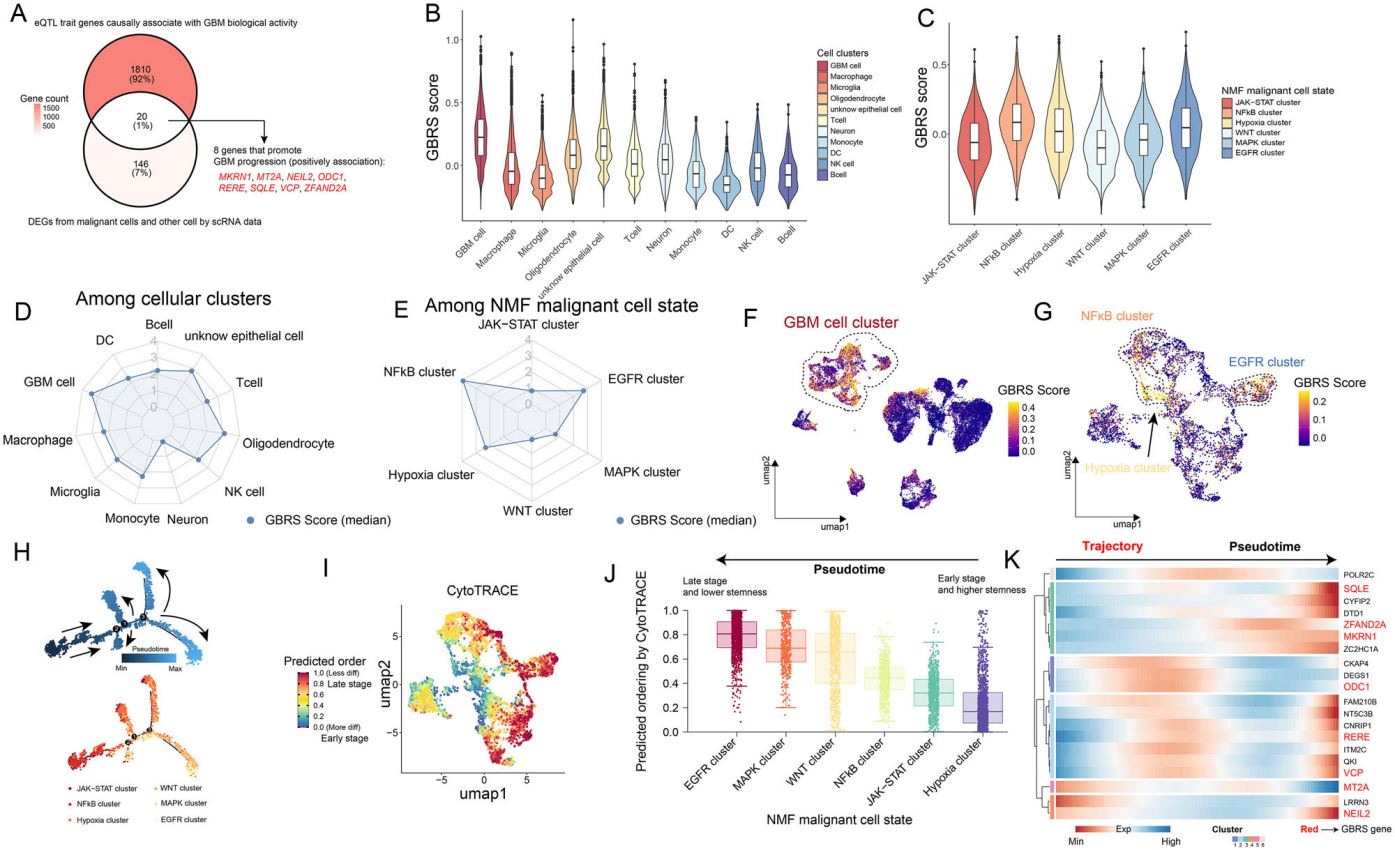
GBRS construction and the characteristics. (A) From the overlap genes originating from eQTL and scRNA‐seq findings, we selected eight markers to establish the GBRS. (B–E) GBRS score calculated by AddModuleScore function in Seurat among 11 available cell types (B, D) and 6 GBM cell states (C, E). The GBRS score was higher in GBM cells and in NFκB, EGFR and Hypoxia clusters. (F–G) From UMAP feature plots, the GBRS score was enriched in the GBM cell cluster (F), NFκB, EGFR, and Hypoxia clusters (G). (H) Semisupervised pseudotime trajectory of GBM cell states inferred by Monocle2. Trajectory was colored by pseudotime (top), and GBM cell states were identified by NMF (bottom). (I–J) CytoTRACE results suggested differentiation status and stemness. Unlike immune cells or stromal cells, the more aggressive malignant cells always have less differentiation and state in the late developmental stage. We could observe the EGFR cluster was in a dramatically less differentiated status (I) and in the very late pseudotemporal stage with lower stemness (J). All the findings elucidated that the EGFR cluster seemed to be more aggressive and poor‐prognosis‐relevant among six GBM cell states. (K) The pseudotime heatmap confirmed almost all GBRS markers were highly expressed in the late stage of trajectory. Thus, the GBRS could mostly pinpoint the aggressiveness of malignant cells. DEGs, differentially expressed genes; GBRS, GBM risk signature.

From eQTL data, we identified 1830 eQTL trait genes causally associated with GBM biological activity (Table S21), and then after differential expression analysis between GBM cells and other cell types, 166 DEGs highly enriched in malignant cells were identified (Table S22, Figure S4G). Among 20 overlapping genes, we selected 8 that were positively associated with GBM progression and proliferation: *MKRN1*, *MT2A*, *NEIL2*, *ODC1*, *RERE*, *SQLE*, *VCP*, and *ZFAND2A* as GBRS markers (Figure 6A, Figure S5). In the scRNA‐seq dataset, we calculated the AddModuleScore as the GBRS score with the eight markers; the GBRS score was the highest in the GBM cell cluster across all malignant as well as nonmalignant clusters (Figure 6B, Table S23). For malignant cell states derived from NMF analysis, the GBRS score was the highest in the NFκB cluster, followed by the EGFR cluster and hypoxia cluster (Figure 6C, Table S24). The median GBRS score was also illustrated in satellite plots across all GBM clusters and malignant cell states (Figure 6D,E). In feature plots, it was also evident that the GBM cell cluster had a higher GBRS score (Figure 6F), and the NFκB cluster EGFR cluster, and the hypoxia cluster obtained a profound GBRS score over other malignant cell states (Figure 6G). These findings indicate that the NFκB cluster and EGFR cluster exhibit heightened lethality and aggressiveness among the six‐cell states, consistent with prior functional analyses that suggest their role in regulating gliogenesis, neurogenesis, and glial cell differentiation (Figure 5G). Pseudotime trajectory analysis revealed the presence of three lineage nodes and seven developmental branches (Figure S6A). Analysis of the pseudotime and pseudotemporal trajectory revealed a differentiation sequence from Node 2 to Node 1 to Node 3. Most cells from the hypoxia and JAK‐STAT clusters were situated in the early stage of the overall trajectory, while the majority of EGFR cluster cells were positioned in the late stage (Figure 6I). In contrast to immune cells or stromal cells, malignant cells exhibit a pronounced proliferation phenotype over differentiation. It is widely recognized that more aggressive malignant cells typically exhibit reduced differentiation, increased proliferation, and reside in the late cell lineage, and vice versa. To validate the findings of the pseudotime trajectory, CytoTRACE results demonstrated that the EGFR cluster exhibited reduced differentiation, lower stemness, and resided in the late stage, whereas the hypoxia cluster displayed increased differentiation, higher stemness, and resided in the early stage (Figure 6J,K). Additionally, we depicted the expression changes of the eight GBRS markers over pseudotime, revealing that all markers exhibited higher expression in the late pseudotime stage compared to the early stage, except for *ODC1* (Figure 6K). This observation is consistent with previous findings indicating that GBRS markers are highly expressed in more malignant cell states (e.g., EGFR cluster) and in the late cell trajectory lineage (Figure 6E, Figure S7). Furthermore, as pseudotime progresses, the GBRS score generally increases, indicating a positive correlation between the GBRS score and the aggressiveness of GBM cells (Figure S6B).

### 3.5 Targeted Drugs for GBRS in Treating GBM

**FIGURE 7:**
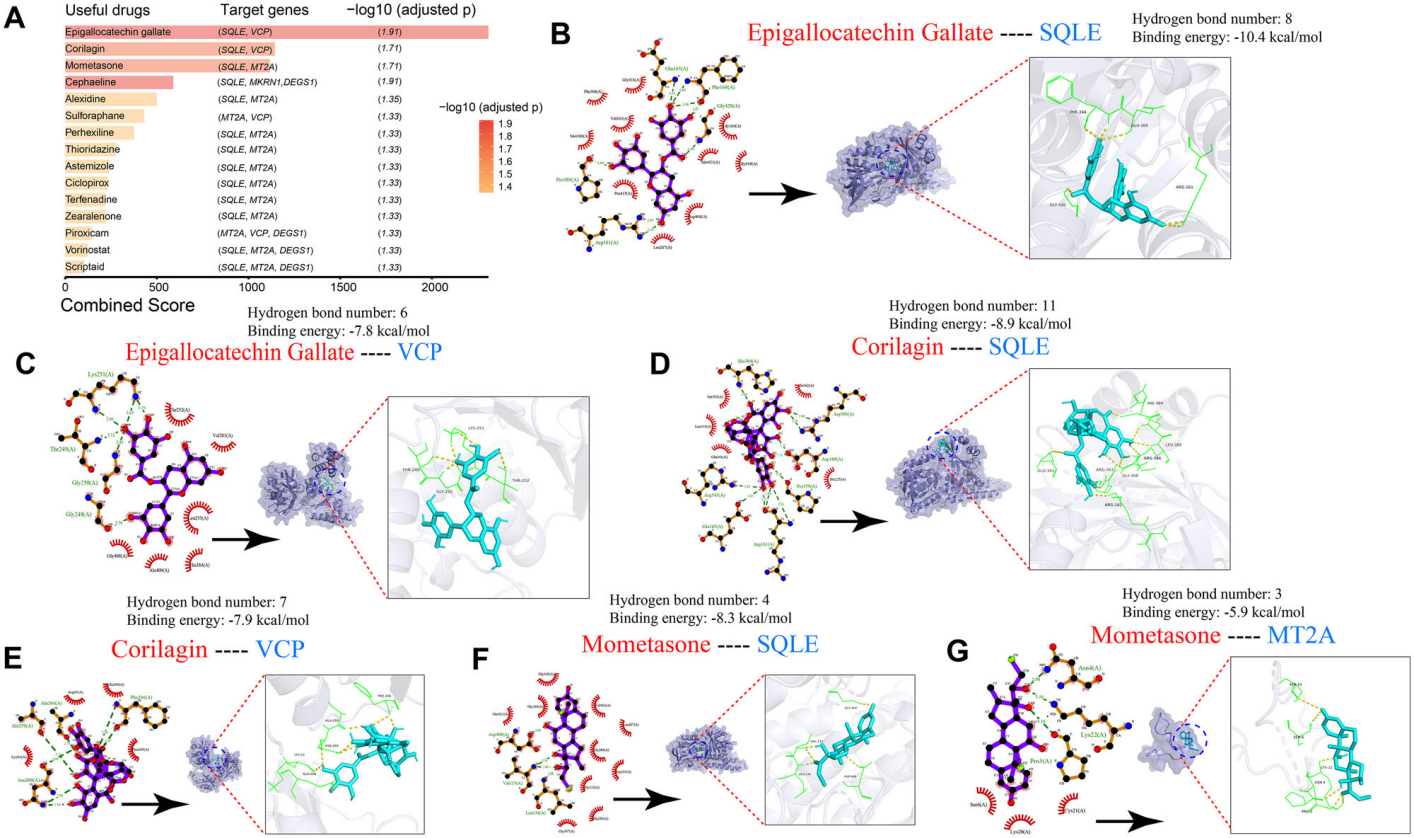
Targeted compounds against GBM via GBRS markers. (A) The top 15 targeted compounds identified by Enrichr are listed in ascending order according to their combined score. EGCG, corilagin, and mometasone emerged as the top three compounds with the highest combined scores. (B–G) Molecular docking profiles between proteins and compounds, including hydrogen bond number and binding energy, were visualized. Specifically, we focused on dockings involving EGCG, corilagin, and mometasone with the GBRS markers *SQLE*, *VCP*, and *MT2A*, as these three compounds exhibited the highest combined scores. EGCG, epigallocatechin gallate.

Using the DSigDB database from the Enrichr website, we conducted a search for potentially efficacious interventional drugs targeting the GBRS markers. The top 15 potential chemical compounds, identified based on their combined score and adjusted *p* value were identified (Figure 7A). Epigallocatechin gallate (EGCG), corilagin, and mometasone emerged as the top three compounds with the highest combined scores (2308, 1144, and 1116, respectively), targeting *SQLE*, *VCP*, and *MT2A* (Table S25). We further delved into how these three compounds exert their protective effects via the targeted markers in GBM. The binding interactions between the top three compounds and the targeted markers were examined using MD (Figure 7B–G). The validation of MD confirmed that the relative binding energies fell within the suitable range for the interaction of the three compounds. Specifically, *SQLE* exhibited the lowest binding energy with EGCG (−10.4 kcal/mol) (Figure 7B) and the highest number of hydrogen bonds, 11, with corilagin (Figure 7D), while *MT2A*‐mometasone demonstrated the highest binding energy (−5.9 kcal/mol) (Figure 7G). Typically, lower binding energy and a higher number of hydrogen bonds indicate a more stable MD.

## 4 Discussion

This study revealed the multifaceted roles of ICTs in the pathogenesis or prevention of 13 brain disorders, with individual ICTs exhibiting divergent functional profiles across diseases. GBM, distinguished by its refractory nature, immunologically cold TME, marked intratumoral heterogeneity, and limited therapeutic efficacy, remains underexplored within neuroscience. Leveraging scRNA‐seq datasets, we classified GBM cells into six distinct states, each characterized by unique biological functions. Furthermore, we developed an eight‐marker GBRS that demonstrates a causal positive association with GBM progression. To identify targeted therapeutics against the GBRS, EGCG emerged as the most promising candidate, exerting protective effects primarily through the *SQLE* and *VCP* markers, with specificity for the EGFR‐driven cell state. This investigation represented the largest MR study to date, elucidating the associations between ICTs and a broad spectrum of brain disorders. Additionally, we established a novel GBRS causally linked to GBM progression using integrated scRNA‐seq and eQTL datasets while identifying potential therapeutic agents. These findings highlighted the pivotal role of neuroinflammation in the etiology of brain disorders and provided novel insights into the molecular characteristics and therapeutic management of GBM.

Recent decades have witnessed significant advances in understanding neuroinflammation within the CNS, particularly its role in disrupting CNS structure and function through pathogenic immune responses (Shabab et al. 2017). Neuroinflammation is a critical driver in the onset and progression of inflammation‐associated neurological disorders, including MS, ALS, AD, PD, and GBS, as well as other conditions not traditionally classified as inflammatory, such as stroke, dementia, and epilepsy (Heneka et al. 2015; Ransohoff 2016). A hallmark of neuroinflammation is leukocyte recruitment, driven by microglial activation in response to exogenous and endogenous stimuli. This activation triggers intracellular signaling pathways, including phosphoinositide 3‐kinase/protein kinase B (PI3K/AKT), mitogen‐activated protein kinase (MAPK), and mammalian target of rapamycin (mTOR), leading to the production of pro‐inflammatory cytokines and chemokines that amplify the neuroinflammatory cascade (Cianciulli et al. 2020; Jadhav et al. 2014). In AD, microglia, the brain's resident immune cells, exhibit a dual role. Under physiological conditions, microglia clear debris and maintain homeostasis; however, in AD, they become dysregulated, releasing pro‐inflammatory cytokines such as tumor necrosis factor‐α (TNF‐α) and interleukin‐1β (IL‐1β). These cytokines exacerbate neuroinflammation, accelerating neuronal damage and amyloid‐β plaque formation, a hallmark of AD pathology (Leng and Edison 2021; Han et al. 2021). Additionally, the inflammatory microenvironment impairs synaptic function, directly contributing to disease progression. Targeting microglial activation and inhibiting pro‐inflammatory pathways represent promising therapeutic strategies for neurodegenerative disorders. Other cytokines, including IL‐6, IL‐18, IL‐12, IL‐23, and IL‐33, are implicated in neurological and neuropsychiatric disorders, shaping the inflammatory TME and influencing pathogenesis in CNS disorders, aging, and tumorigenesis (Becher et al. 2017).

In cancer, the immune TME promotes tolerance, facilitating tumor survival and progression. M2‐like tumor‐associated macrophages (TAMs) secrete IL‐10 and growth factors that enhance tumor progression, angiogenesis, and metastasis while suppressing cytotoxic T‐cell responses, thus enabling immune evasion (Li et al. 2023). This immunosuppression is further reinforced by programmed cell death ligand 1 (PD‐L1) expression on tumor cells (Yi et al. 2022). Reprogramming TAMs to bolster antitumor immunity represents a potential therapeutic avenue. In contrast to their destructive role in neurological disorders, immune cells in cancer exacerbate immune evasion and tumor aggression. In summary, immune cells drive deleterious inflammation in neurological disorders while promoting immune tolerance and tumor progression in cancer. These insights, leveraged by the current study, underscore the therapeutic potential of immune modulation for managing CNS disorders and malignancies.

Single‐cell methodologies, particularly scRNA‐seq, have revolutionized the characterization of the GBM TME, revealing unparalleled cellular diversity and niche complexity (Karimi et al. 2023; Louis et al. 2021). Unlike other malignancies, GBM exhibits a distinct, immunologically cold TME characterized by functionally diverse astrocytes, pro‐tumorigenic macrophages, and a paucity of infiltrating lymphocytes. The primary barriers to effective therapy include pronounced intratumoral heterogeneity, which integrative approaches—combining transcriptional and genetic profiling—can elucidate by clarifying the interplay between genetic alterations and epigenetic state diversity (Neftel et al. 2019). Historically, bulk RNA‐seq data stratified GBM into three major subtypes: proneural (TCGA‐PN), classical (TCGA‐CL), and mesenchymal (TCGA‐MES). However, these subtypes often coexist within a single tumor, with regional variation and dynamic shifts in response to therapy or over time (Verhaak et al. 2010; Wang et al. 2017). Another study classified GBM cells into distinct states—NPC‐like, OPC‐like, AC‐like, and MES‐like—based on single‐cell RNA‐seq data, though their lineage trajectories and prognostic implications remain unresolved (Neftel et al. 2019). Personalized GBM therapy requires targeting specific cell states to overcome heterogeneity‐driven resistance. In this context, EGCG emerged as a promising therapeutic candidate, targeting *SQLE* and *VCP*, which were highly expressed in the EGFR‐driven cluster. *SQLE* is also an unfavorable marker for pancreatic cancer (*p *< 0.001), lung cancer (*p *< 0.001), renal cell carcinoma (*p *< 0.001), and head and neck cancer (*p *< 0.001); *VCP* also shows unfavorable prognostic roles in lung cancer (*p *= 0.011), liver cancer (*p *= 0.003), and melanoma (*p *= 0.038) by the Human Protein Atlas project (https://www.proteinatlas.org/). EGCG is the primary catechin in green tea, exhibiting potential anticancer effects in both in vivo and in vitro. Mechanistically, EGCG inhibits cancer cell proliferation by inducing cell cycle arrest at G0/G1 or G2/M and promotes apoptosis by activating intrinsic (Bax/Bcl‐2, Caspase‐3/9) and extrinsic (Fas, TRAIL) pathways, often in a p53‐dependent manner. Additionally, EGCG inhibits invasion and metastasis by suppressing matrix metalloproteinases (MMP‐2/9) and reversing epithelial–mesenchymal transition (EMT) through TGF‐β/Smad inhibition; also angiogenesis is restricted by binding vascular endothelial growth factor (VEGF) and inhibiting HIF‐1α. M1‐like TAM polarization and CD8T cell activation could be promoted, while Tregs are suppressed to elicit more antitumor immunity. Besides, cancer stem cells (CSCs) can be targeted by EGCG to reduce cancer stemness, and the exerted antioxidant effects reactivated tumor suppressor genes. Finally, EGCG sensitizes tumor response to chemotherapy and radiotherapy (Liu et al. 2024; Y. Zhang et al. 2015; Almatroodi et al. 2020). However, given the limited bioactivity and high metabolism efficiency of EGCG, the real translation into clinical benefits warrants further investigation.

This study had several limitations that warrant consideration for future research. Although scRNA‐seq provided profound insights into cellular heterogeneity, its ability to capture cell surface markers was constrained, limiting the validation of numerous ICTs using transcriptomic data alone. The integration of CITE‐seq will offer a promising approach to overcome these limitations by enabling simultaneous evaluation of surface markers and transcriptomic profiles, thereby enhancing the characterization of ICTs. Secondly, the MR analyses were limited by the small sample sizes of some GWAS datasets included in this study. Reduced sample sizes diminished statistical power, potentially yielding imprecise causal estimates and increasing the risk of bias due to population‐specific effects or sampling variability. Furthermore, small disease case numbers restricted the feasibility of robust sensitivity analyses to address potential violations of MR assumptions, such as pleiotropy or heterogeneity. These constraints necessitated a cautious interpretation of the findings and underscored the need for larger, more diverse cohorts to validate and strengthen the results. Additionally, the large‐scale cohorts used in the MR analyses lacked comprehensive demographic data, precluding subgroup‐specific analyses based on clinical parameters. Given the heterogeneity of subtypes within certain brain diseases, extrapolating these findings to clinical practice required caution. The increasing availability of single‐cell and spatial transcriptomic tools presents opportunities to further explore ICTs across nonmalignant and malignant brain disorders. Of particular interest is whether ICTs with causal effects exhibit distinct functional attributes in specific anatomical regions, a question that spatial transcriptomics could address in future investigations.

## 5 Conclusion

This study elucidated the causal roles of ICTs in 13 malignant and nonmalignant CNS disorders, highlighting the multifaceted yet critical contribution of neuroinflammation to their etiology. Among six GBM cell states identified through scRNA‐seq, the NFκB‐ and EGFR‐driven clusters exhibited heightened aggressiveness, characterized by elevated GBRS. eQTL‐derived GBRS demonstrated a causal association with GBM incidence. Furthermore, EGCG emerged as a promising therapeutic candidate, targeting SQLE and VCP within the EGFR‐driven cluster. These findings established causal links between ICTs and CNS disorders, enhanced scientific understanding of neuroinflammatory mechanisms, and provided novel insights into GBM heterogeneity, molecular pathogenesis, and targeted therapeutic strategies.

## Author Contributions

**Shanbao Ke**: conceptualization, methodology, software, data curation, formal analysis, writing–original draft, investigation, validation, visualization. **Junya Yan**: software, formal analysis, project administration, supervision, writing–original draft. **Baiyu Li**: writing–review and editing, writing–original draft, supervision, visualization, resources. **Xiao Feng**: writing–original draft, visualization, project administration, resources, writing–review and editing, supervision.

## Conflicts of Interest

The authors declare no conflicts of interest.

## Peer Review

The peer review history for this article is available at https://publons.com/publon/10.1002/brb3.70632

## Supporting information




**Figure S1**. **The MR results for CD45 on CD33^bright^ HLA DR^+^ CD14^−^ myeloid cell ICT on GBM**. (**A**) Scatter plots revealed consistent findings across five MR methods, indicating this ICT could reduce the risk for GBM. (**B**) Meta‐analyses with 19 IVs in MR‐Egger and IVW methods showed the protection role of this ICT. (**C**–**D**) Funnel plot and leave‐one‐out analysis addressed the robustness of these results. ICT, immune cell trait; GBM, glioblastoma; IVW method, inverse variance weighted method.

**Figure S2**. **The MR results for CD3 on CD39^+^ resting Tregs ICT on GBM**. (**A**) Scatter plots displayed consistent findings across five MR methods, indicating this ICT could reduce the risk for GBM. (**B**) Meta‐analyses with 19 IVs in MR‐Egger and IVW methods showed the impact of reduced risk derived from this ICT on GBM. (**C**–**D**) Funnel plot and leave‐one‐out analysis addressed the robustness of these results.

**Figure S3. CNV events evaluated by inferCNV**. The InferCNV algorithm was utilized to understand the real malignant cellular clusters. In this case, it helped to ensure that the AC‐like, NPC‐like, OPC‐like, and MES‐like clusters were malignant cells. Of note, not all but some unknown epithelial cells were also malignant cells.

**Figure S4. Functional analyses results on metaGene of each cell state and DEGs among 11 available cell types. (A‐F)** GO enrichment results for the metaGene in the JAK‐STAT state (**A**), NK‐κB state (**B**), hypoxia state (**C**), WNT state (**D**), MAPK state (**E**), and EGFR state (**F**), respectively. (**G**) The heatmap showed DEGs and representative marker genes (on the left) among 11 available cell types from scRNA‐seq. GO, gene ontology; DEGs, differentially expressed genes.

**Figure S5. Scatter plots for GBRS makers. (A‐H)** Based on scRNA‐seq and eQTL data, we constructed an 8‐marker GBRS that was associated with GBM progression. The scatter plots just confirmed the positive association between MKRN1 (**A**), MT2A (**B**), NEIL2 (**C**), ODC1 (**D**), RERE (**E**), SQLE (**F**), VCP (**G**), ZFAND2A (**H**), and GBM progression. All five MR methods exhibited consistent results. GBRS, GBM risk signature.

**Figure S6. Changes of GBRS score with pseudotime. (A)** Semisupervised pseudotime trajectory of GBM cell states inferred by Monocle2. The trajectory was color‐coded according to seven states that mainly emerged at distinct developmental stages identified by Monocle2, which differed from the GBM cell states identified by NMF. (**B**) The GBRS score exhibited an increase with pseudotime going, indicating a positive association between GBRS score and the aggressiveness of GBM cells.

**Figure S7. Relative expression of 8 markers of GBRS in 6 GBM cell states**. The cell state enclosed by a frame exhibited the highest expression of each marker compared to the other states.

STROBE‐MR Statement Checklist

**Table S1**. Summarization of GWAS data sources for 731 ICTs on 13 brain diseases.
**Table S2**. The introduction of 731 ICTs.
**This is the graph abstract of our manuscript delineating the methods employed in both MR and scRNA‐seq analyses within the present investigationTable S3**. MR study results of 731 ICTs on ischemic stroke.
**Table S4**. MR study results of 731 ICTs on multiple sclerosis.
**Table S5**. MR study results of 731 ICTs on Guillain–Barre syndrome.
**Table S6**. MR study results of 731 ICTs on Parkinson's disease.
**Table S7**. MR study results of 731 ICTs on amyotrophic lateral sclerosis.
**Table S8**. MR study results of 731 ICTs on epilepsy.
**Table S9**. MR study results of 731 ICTs on dementia.
**Table S10**. MR study results of 731 ICTs on Alzheimer's disease.
**Table S11**. MR study results of 731 ICTs on myasthenia gravis.
**Table S12**. MR study results of 731 ICTs on glioma.
**Table S13**. MR study results of 731 ICTs on glioblastoma.
**Table S14**. MR study results of 731 ICTs on major depressive disorder.
**Table S15**. MR study results of 731 ICTs on schizophrenia.
**Table S16**. CellChat results for all interactions among 11 available cell types.
**Table S17**. LIANA results for all interactions among 11 available cell types.
**Table S18**. Representative genes in 6 identified metaprograms.
**Table S19**. MetaGenes (top 25) in 6 identified metaprograms.
**Table S20**. GO functional analyses results for the metaGene in each metaprogram.
**Table S21**. MR study results of eQTL paired genes on glioblastoma.
**Table S22**. Differentially expressed genes among 11 available cell types in the scRNA‐seq dataset.
**Table S23**. GBRS score in 11 cell types by scRNA‐seq.
**Table S23**. GBRS score in 6 identified cell states by scRNA‐seq.
**Table S24**. GBRS score in 6 identified cell states by scRNA‐seq.
**Table S25**. Top 15 targeted compounds results by Enrichr and selection procedure.

## Data Availability

Data sharing not applicable to this article as no datasets were generated or analyzed during the current study.
