# Supplementary figures and images for: Causal Association Between Immune Cell Traits and Risk of Multiple Malignant and Nonmalignant CNS Diseases: A Mendelian Randomization and Single‐Cell Transcriptomic Analysis
Source: Brain Behav. 2025 Aug 4;15(8):e70632. doi: 10.1002/brb3.70632 (PMC12321959; doi:10.1002/brb3.70632)

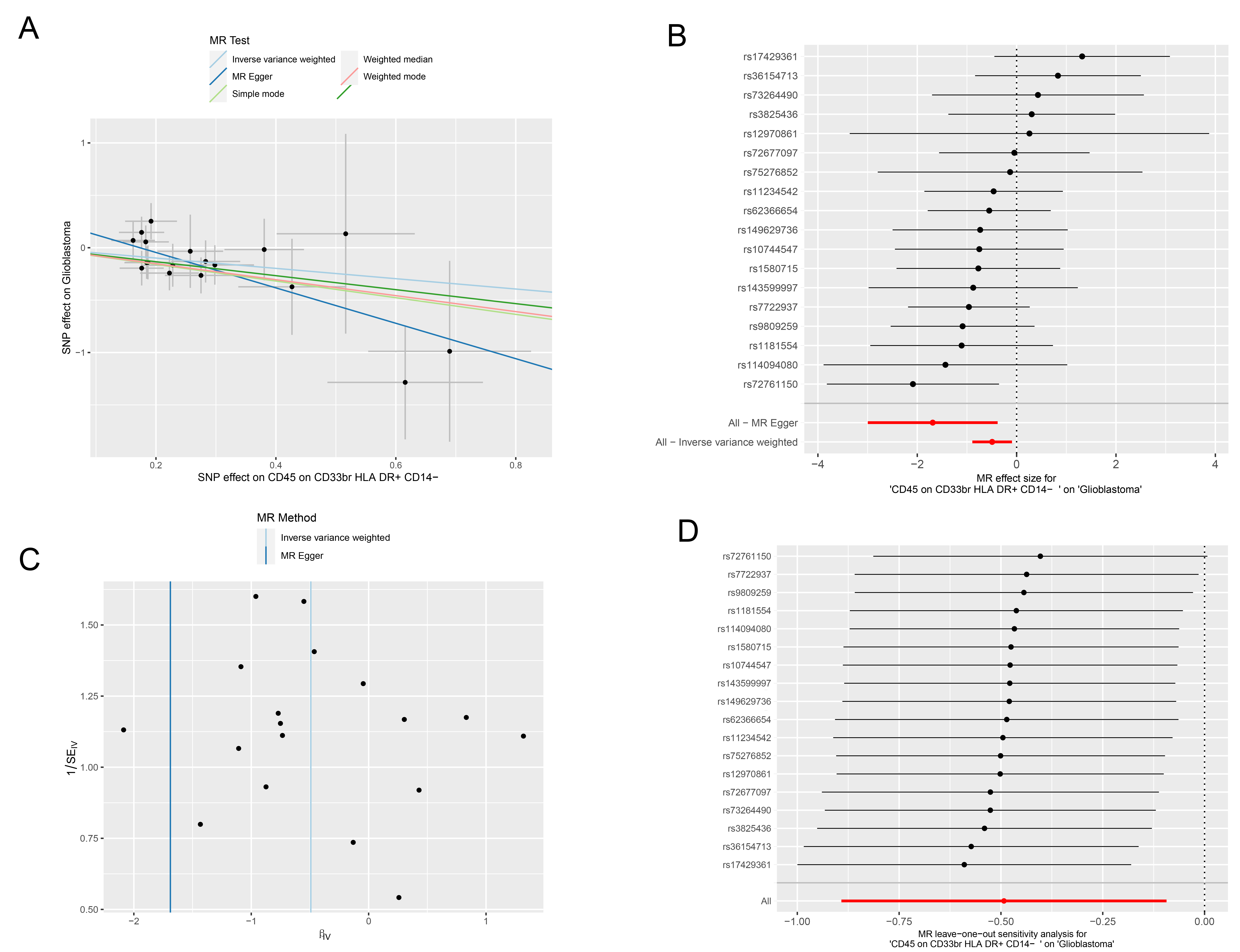

Supplement: Supplementary file 1 — Figure S1. The MR results for CD45 on CD33bright HLA DR+ CD14− myeloid cell ICT on GBM. (A) Scatter plots revealed consistent findings across five MR methods, indicating this ICT could reduce the risk for GBM. (B) Meta‐analyses with 19 IVs in MR‐Egger and IVW methods showed the protection role of this ICT. (C–D) Funnel plot and leave‐one‐out analysis addressed the robustness of these results. ICT, immune cell trait; GBM, glioblastoma; IVW method, inverse variance weighted method. [file BRB3-15-e70632-s006.tif]

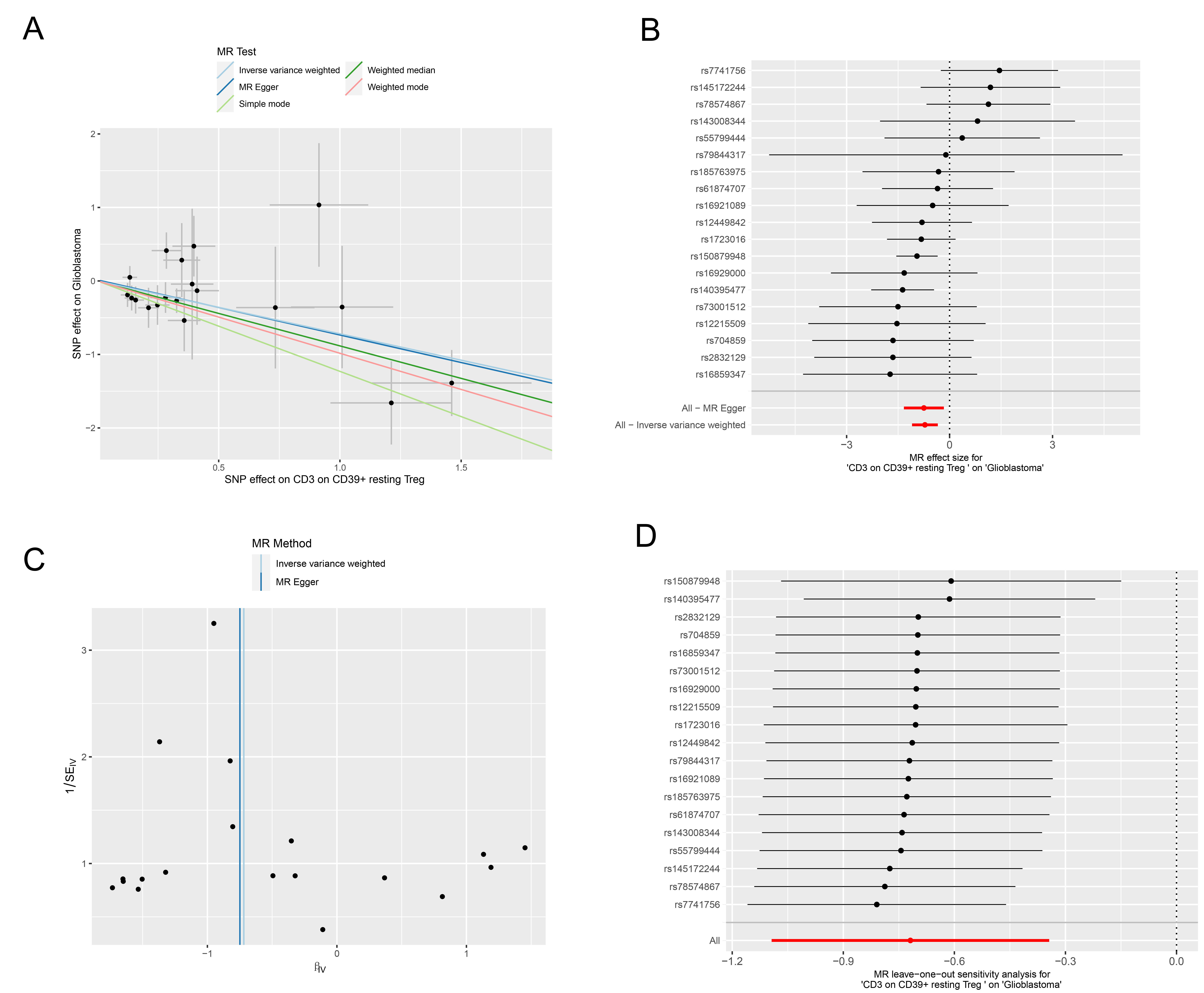

Supplement: Supplementary file 2 — Figure S2. The MR results for CD3 on CD39+ resting Tregs ICT on GBM. (A) Scatter plots displayed consistent findings across five MR methods, indicating this ICT could reduce the risk for GBM. (B) Meta‐analyses with 19 IVs in MR‐Egger and IVW methods showed the impact of reduced risk derived from this ICT on GBM. (C–D) Funnel plot and leave‐one‐out analysis addressed the robustness of these results. [file BRB3-15-e70632-s005.tif]

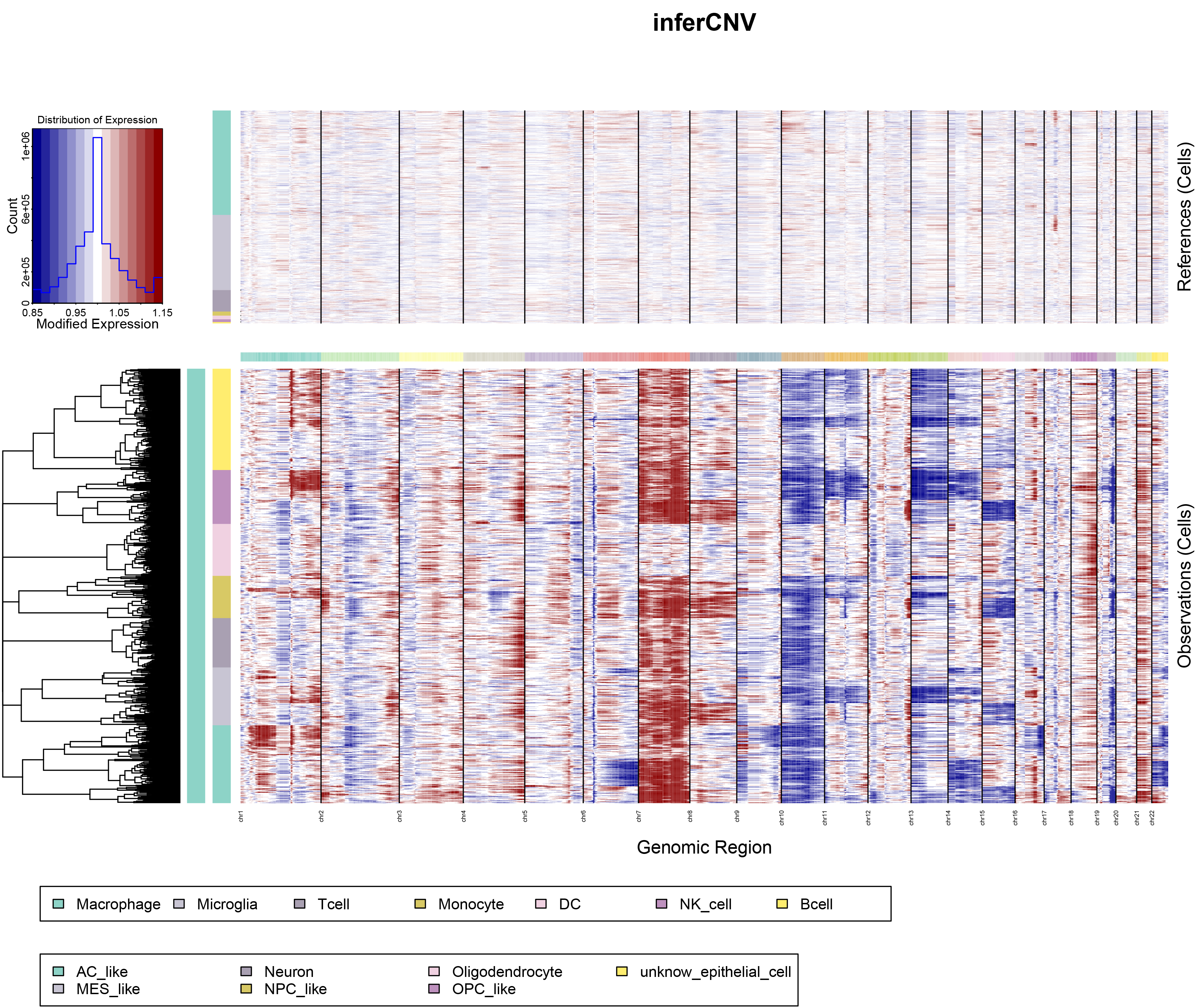

Supplement: Supplementary file 3 — Figure S3. CNV events evaluated by inferCNV. The InferCNV algorithm was utilized to understand the real malignant cellular clusters. In this case, it helped to ensure that the AC‐like, NPC‐like, OPC‐like, and MES‐like clusters were malignant cells. Of note, not all but some unknown epithelial cells were also malignant cells. [file BRB3-15-e70632-s001.tif]

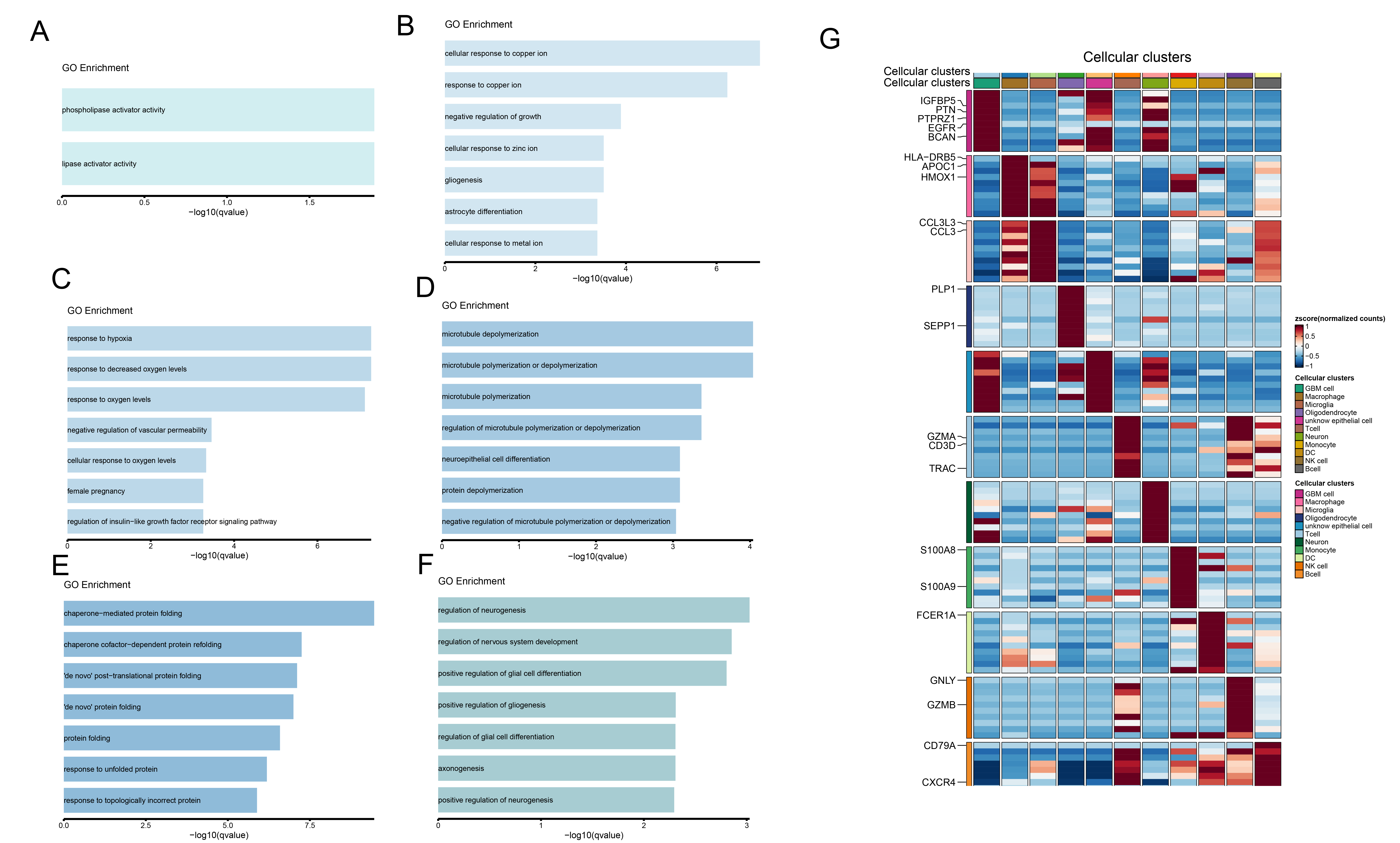

Supplement: Supplementary file 4 — Figure S4. Functional analyses results on metaGene of each cell state and DEGs among 11 available cell types. (A‐F) GO enrichment results for the metaGene in the JAK‐STAT state (A), NK‐κB state (B), hypoxia state (C), WNT state (D), MAPK state (E), and EGFR state (F), respectively. (G) The heatmap showed DEGs and representative marker genes (on the left) among 11 available cell types from scRNA‐seq. GO, gene ontology; DEGs, differentially expressed genes. [file BRB3-15-e70632-s003.tif]

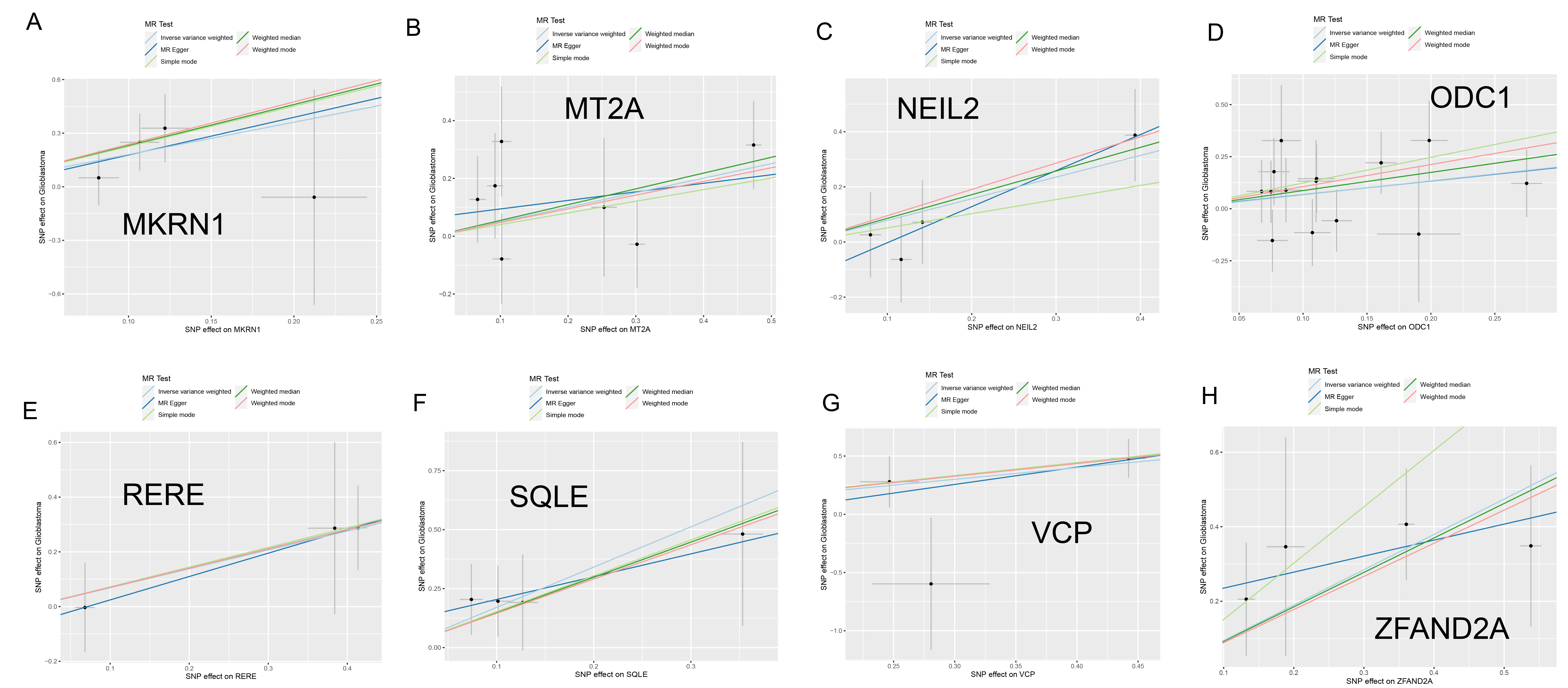

Supplement: Supplementary file 5 — Figure S5. Scatter plots for GBRS makers. (A‐H) Based on scRNA‐seq and eQTL data, we constructed an 8‐marker GBRS that was associated with GBM progression. The scatter plots just confirmed the positive association between MKRN1 (A), MT2A (B), NEIL2 (C), ODC1 (D), RERE (E), SQLE (F), VCP (G), ZFAND2A (H), and GBM progression. All five MR methods exhibited consistent results. GBRS, GBM risk signature. [file BRB3-15-e70632-s007.tif]

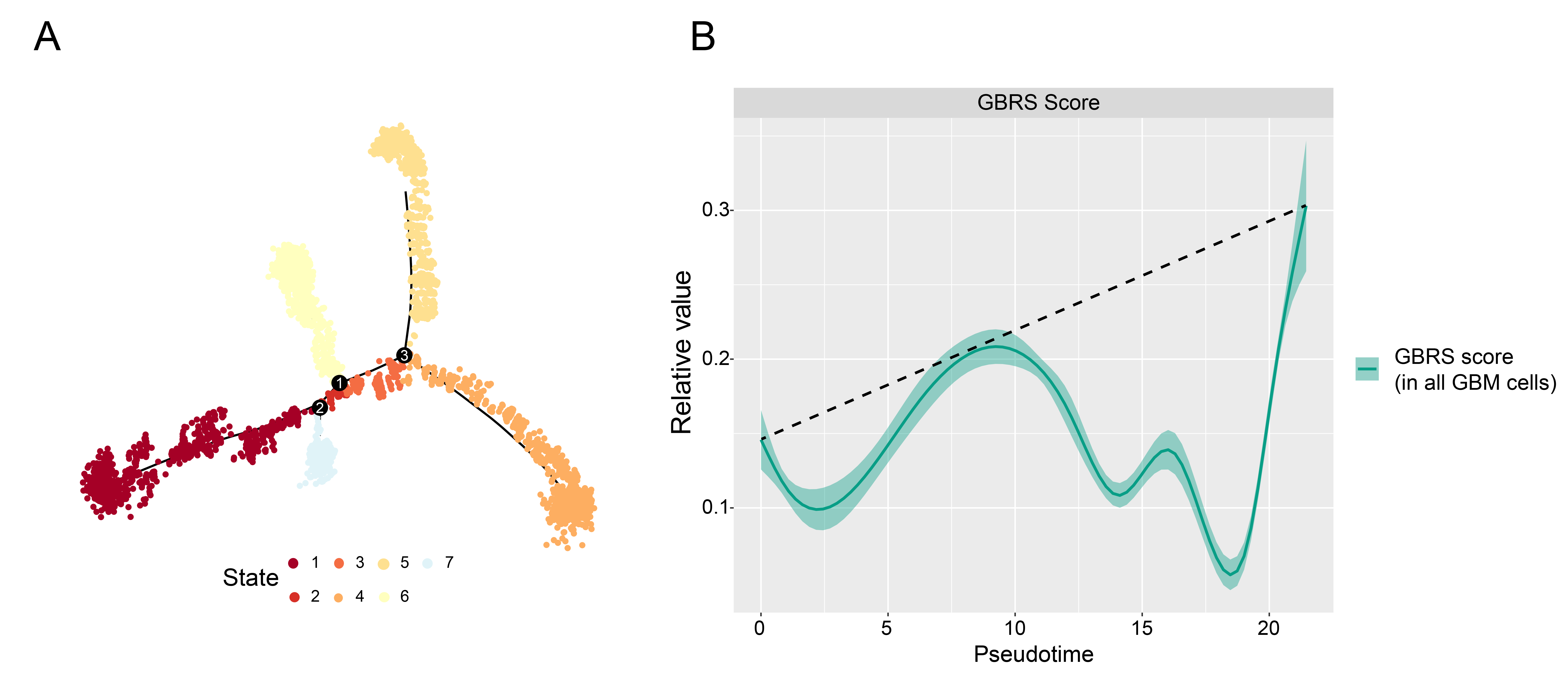

Supplement: Supplementary file 6 — Figure S6. Changes of GBRS score with pseudotime. (A) Semisupervised pseudotime trajectory of GBM cell states inferred by Monocle2. The trajectory was color‐coded according to seven states that mainly emerged at distinct developmental stages identified by Monocle2, which differed from the GBM cell states identified by NMF. (B) The GBRS score exhibited an increase with pseudotime going, indicating a positive association between GBRS score and the aggressiveness of GBM cells. [file BRB3-15-e70632-s008.tif]

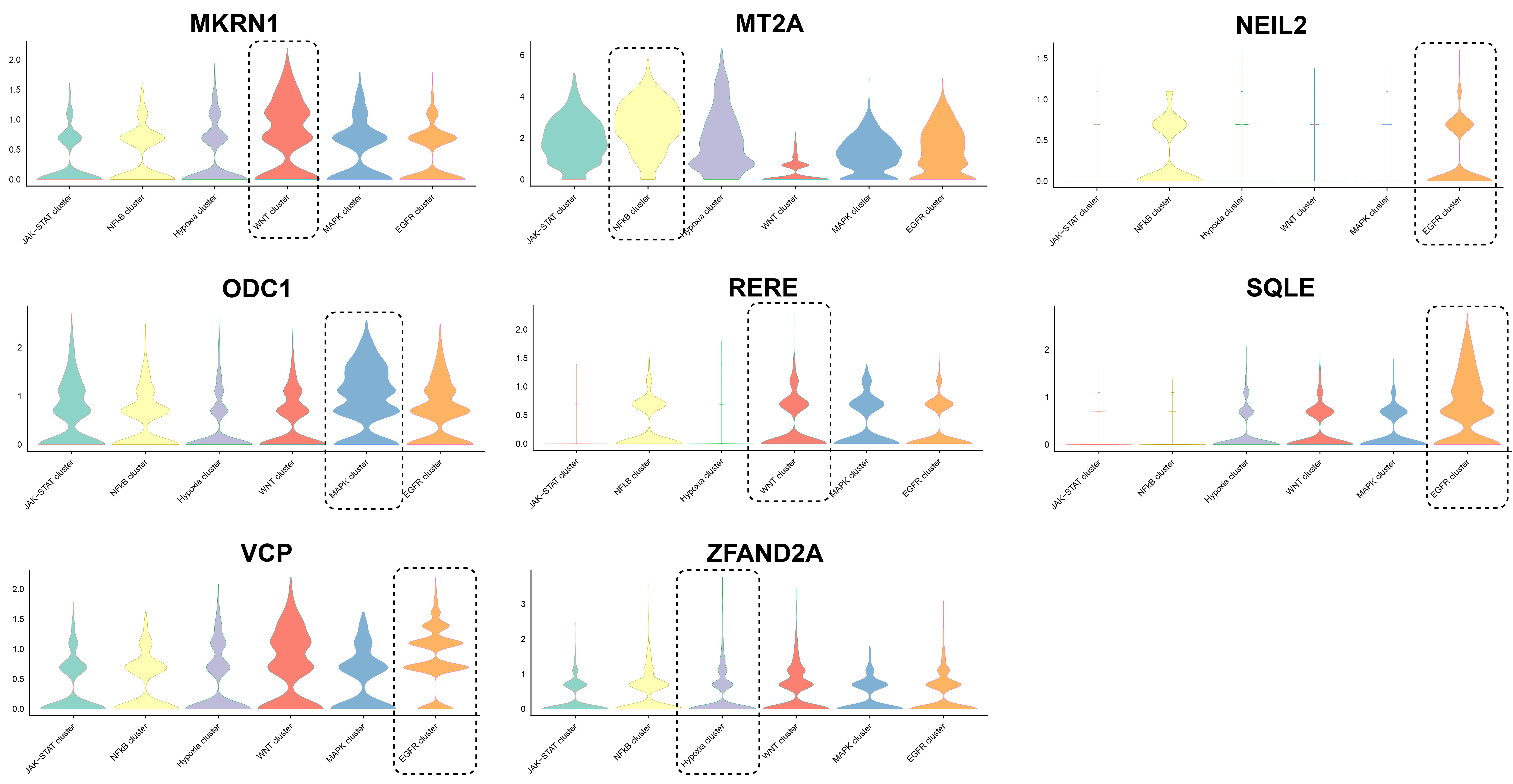

Supplement: Supplementary file 7 — Figure S7. Relative expression of 8 markers of GBRS in 6 GBM cell states. The cell state enclosed by a frame exhibited the highest expression of each marker compared to the other states. [file BRB3-15-e70632-s002.tif]
