# Supplementary material for: Causal Association Between Immune Cell Traits and Risk of Multiple Malignant and Nonmalignant CNS Diseases: A Mendelian Randomization and Single‐Cell Transcriptomic Analysis
Source: Brain Behav. 2025 Aug 4;15(8):e70632. doi: 10.1002/brb3.70632 (PMC12321959; doi:10.1002/brb3.70632)
Supplement: Supplementary file 8 — STROBE‐MR Statement Checklist [file BRB3-15-e70632-s009.docx]

**STROBE-MR checklist of recommended items to address in reports of Mendelian randomization studies**^1^ ^2^

| **Item No.** | **Section** | **Checklist item** | **Page No.** | **Relevant text from manuscript** |
| --- | --- | --- | --- | --- |
| 1 | **TITLE and ABSTRACT** | Indicate Mendelian randomization (MR) as the study’s design in the title and/or the abstract if that is a main purpose of the study | 1, 3 | See the manuscript |
|  | **INTRODUCTION** |  | 4-6 |  |
| 2 | **Background** | Explain the scientific background and rationale for the reported study. What is the exposure? Is a potential causal relationship between exposure and outcome plausible? Justify why MR is a helpful method to address the study question | 4-5 | See the manuscript |
| 3 | **Objectives** | State specific objectives clearly, including pre-specified causal hypotheses (if any). State that MR is a method that, under specific assumptions, intends to estimate causal effects | 5-6 | See the manuscript |
|  | **METHODS** |  | 6-17 |  |
| 4 | **Study design and data sources** | Present key elements of the study design early in the article. Consider including a table listing sources of data for all phases of the study. For each data source contributing to the analysis, describe the following: | 6-7 | See the manuscript and Table S1 |
|  | a) | Setting: Describe the study design and the underlying population, if possible. Describe the setting, locations, and relevant dates, including periods of recruitment, exposure, follow-up, and data collection, when available. | 6-7 | See the manuscript and Table S1 |
|  | b) | Participants: Give the eligibility criteria, and the sources and methods of selection of participants. Report the sample size, and whether any power or sample size calculations were carried out prior to the main analysis | 6-7 | See the manuscript and Table S1 |
|  | c) | Describe measurement, quality control and selection of genetic variants | 7-8 | See the manuscript |
|  | d) | For each exposure, outcome, and other relevant variables, describe methods of assessment and diagnostic criteria for diseases | 6-7 | See the manuscript |
|  | e) | Provide details of ethics committee approval and participant informed consent, if relevant | 16-17 | See the manuscript |
| 5 | **Assumptions** | Explicitly state the three core IV assumptions for the main analysis (relevance, independence and exclusion restriction) as well assumptions for any additional or sensitivity analysis | 7-8 | See the manuscript |
| 6 | **Statistical methods: main analysis** | Describe statistical methods and statistics used | 8-9 | See the manuscript |
|  | a) | Describe how quantitative variables were handled in the analyses (i.e., scale, units, model) | 8-9 | See the manuscript |
|  | b) | Describe how genetic variants were handled in the analyses and, if applicable, how their weights were selected | 8-10 | See the manuscript |
|  | c) | Describe the MR estimator (e.g. two-stage least squares, Wald ratio) and related statistics. Detail the included covariates and, in case of two-sample MR, whether the same covariate set was used for adjustment in the two samples | 8-10 | See the manuscript |
|  | d) | Explain how missing data were addressed | 8-10 | See the manuscript |
|  | e) | If applicable, indicate how multiple testing was addressed | 9-10 | See the manuscript |
| 7 | **Assessment of assumptions** | Describe any methods or prior knowledge used to assess the assumptions or justify their validity | 9-10 | See the manuscript (Colocalization analysis) |
| 8 | **Sensitivity analyses and additional analyses** | Describe any sensitivity analyses or additional analyses performed (e.g. comparison of effect estimates from different approaches, independent replication, bias analytic techniques, validation of instruments, simulations) | 9-10 | See the manuscript (Colocalization analysis) |
| 9 | **Software and pre-registration** |  |  |  |
|  | a) | Name statistical software and package(s), including version and settings used | 8-9 | See the manuscript |
|  | b) | State whether the study protocol and details were pre-registered (as well as when and where) | 8-9 | See the manuscript |
|  | **RESULTS** |  | 17-25 |  |
| 10 | **Descriptive data** |  |  |  |
|  | a) | Report the numbers of individuals at each stage of included studies and reasons for exclusion. Consider use of a flow diagram | 17-19 | See the manuscript |
|  | b) | Report summary statistics for phenotypic exposure(s), outcome(s), and other relevant variables (e.g. means, SDs, proportions) | 17-19 | See the manuscript |
|  | c) | If the data sources include meta-analyses of previous studies, provide the assessments of heterogeneity across these studies | 17-19 | See the manuscript |
|  | d) | For two-sample MR:  i.  Provide justification of the similarity of the genetic variant-exposure associations between the exposure and outcome samples  ii.  Provide information on the number of individuals who overlap between the exposure and outcome studies | 17-19 | See the manuscript |
| 11 | **Main results** |  | 17-19 | See the manuscript |
|  | a) | Report the associations between genetic variant and exposure, and between genetic variant and outcome, preferably on an interpretable scale | 17-19 | See the manuscript |
|  | b) | Report MR estimates of the relationship between exposure and outcome, and the measures of uncertainty from the MR analysis, on an interpretable scale, such as odds ratio or relative risk per SD difference | 17-19 | See the manuscript |
|  | c) | If relevant, consider translating estimates of relative risk into absolute risk for a meaningful time period | 17-19 | See the manuscript |
|  | d) | Consider plots to visualize results (e.g. forest plot, scatterplot of associations between genetic variants and outcome versus between genetic variants and exposure) | 17-19 | See the manuscript |
| 12 | **Assessment of assumptions** |  |  |  |
|  | a) | Report the assessment of the validity of the assumptions | 17-19 | See the manuscript |
|  | b) | Report any additional statistics (e.g., assessments of heterogeneity across genetic variants, such as *I^2^*, Q statistic or E-value) | 17-19 | See the manuscript |
| 13 | **Sensitivity analyses and additional analyses** |  |  |  |
|  | a) | Report any sensitivity analyses to assess the robustness of the main results to violations of the assumptions | 17-19 | See the manuscript |
|  | b) | Report results from other sensitivity analyses or additional analyses | 17-19 | See the manuscript |
|  | c) | Report any assessment of direction of causal relationship (e.g., bidirectional MR) | 17-19 | See the manuscript |
|  | d) | When relevant, report and compare with estimates from non-MR analyses | 17-19 | See the manuscript |
|  | e) | Consider additional plots to visualize results (e.g., leave-one-out analyses) | 17-19 | See the manuscript |
|  | **DISCUSSION** |  | 25-30 |  |
| 14 | **Key results** | Summarize key results with reference to study objectives | 25 | See the manuscript |
| 15 | **Limitations** | Discuss limitations of the study, taking into account the validity of the IV assumptions, other sources of potential bias, and imprecision. Discuss both direction and magnitude of any potential bias and any efforts to address them | 29-30 | See the manuscript |
| 16 | **Interpretation** |  |  |  |
|  | a) | Meaning: Give a cautious overall interpretation of results in the context of their limitations and in comparison with other studies | 25-29 | See the manuscript |
|  | b) | Mechanism: Discuss underlying biological mechanisms that could drive a potential causal relationship between the investigated exposure and the outcome, and whether the gene-environment equivalence assumption is reasonable. Use causal language carefully, clarifying that IV estimates may provide causal effects only under certain assumptions | 25-29 | See the manuscript |
|  | c) | Clinical relevance: Discuss whether the results have clinical or public policy relevance, and to what extent they inform effect sizes of possible interventions | 25-29 | See the manuscript |
| 17 | **Generalizability** | Discuss the generalizability of the study results (a) to other populations, (b) across other exposure periods/timings, and (c) across other levels of exposure | 25-29 | See the manuscript |
|  | **OTHER INFORMATION** |  |  |  |
| 18 | **Funding** | Describe sources of funding and the role of funders in the present study and, if applicable, sources of funding for the databases and original study or studies on which the present study is based | 32 | See the manuscript |
| 19 | **Data and data sharing** | Provide the data used to perform all analyses or report where and how the data can be accessed, and reference these sources in the article. Provide the statistical code needed to reproduce the results in the article, or report whether the code is publicly accessible and if so, where | 32 | See the manuscript |
| 20 | **Conflicts of Interest** | All authors should declare all potential conflicts of interest | 32 | See the manuscript |

This checklist is copyrighted by the Equator Network under the Creative Commons Attribution 3.0 Unported (CC BY 3.0) license.

1. Skrivankova VW, Richmond RC, Woolf BAR, Yarmolinsky J, Davies NM, Swanson SA, et al. Strengthening the Reporting of Observational Studies in Epidemiology using Mendelian Randomization (STROBE-MR) Statement. JAMA. 2021;under review.

2. Skrivankova VW, Richmond RC, Woolf BAR, Davies NM, Swanson SA, VanderWeele TJ, et al. Strengthening the Reporting of Observational Studies in Epidemiology using Mendelian Randomisation (STROBE-MR): Explanation and Elaboration. BMJ. 2021;375:n2233.
